# Merits and pitfalls of conventional and covalent docking in identifying new hydroxyl aryl aldehyde like compounds as human IRE1 inhibitors

**DOI:** 10.1038/s41598-019-39939-z

**Published:** 2019-03-04

**Authors:** Antonio Carlesso, Chetan Chintha, Adrienne M. Gorman, Afshin Samali, Leif A. Eriksson

**Affiliations:** 1 Department of Chemistry and Molecular Biology, University of Gothenburg, 405 30 Göteborg, Sweden; 2 Apoptosis Research Centre, National University of Ireland Galway, Galway, Ireland

## Abstract

IRE1 is an endoplasmic reticulum (ER) bound transmembrane bifunctional kinase and endoribonuclease protein crucial for the unfolded protein response (UPR) signaling pathway. Upon ER stress, IRE1 homodimerizes, oligomerizes and autophosphorylates resulting in endoribonuclease activity responsible for excision of a 26 nucleotide intron from the X-box binding protein 1 (XBP1) mRNA. This unique splicing mechanism results in activation of the XBP1s transcription factor to specifically restore ER stress. Small molecules targeting the reactive lysine residue (Lys907) in IRE1α’s RNase domain have been shown to inhibit the cleavage of XBP1 mRNA. Crystal structures of murine IRE1 in complex with covalently bound hydroxyl aryl aldehyde (HAA) inhibitors show that these molecules form hydrophobic interactions with His910 and Phe889, a hydrogen bond with Tyr892 and an indispensable Schiff-base with Lys907. The availability of such data prompted interest in exploring structure-based drug design as a strategy to develop new covalently binding ligands. We extensively evaluated conventional and covalent docking for drug discovery targeting the catalytic site of the RNase domain. The results indicate that neither computational approach is fully successful in the current case, and we highlight herein the potential and limitations of the methods for the design of novel IRE1 RNase binders.

## Introduction

The unfolded protein response (UPR) is a cellular stress response related to the folding of proteins in the endoplasmic reticulum (ER). It is triggered by the accumulation of misfolded proteins in the luminal domain of the ER. The UPR has two purposes: initially restoring normal cell function by interrupting protein synthesis, and increasing the production of molecular chaperones involved in protein folding. If these goals cannot be restored the UPR initializes apoptosis, a process of programmed cell death^1,2^.

Inositol-requiring enzyme 1 (IRE1), protein kinase RNA (PKR)-like ER kinase (PERK), and activating transcription factor 6 (ATF6) represent the three major arms of the UPR^2^. IRE1 is the most evolutionarily conserved branch of UPR. It is a transmembrane protein with its N-terminal domain in the ER lumen, a single transmembrane helix and a cytoplasmic kinase and ribonuclease domain^3,4^. Under ER stress, IRE1 dimerizes, trans-autophosphorylates and activates its endoribonuclease domain^5,6^. The endoribonuclease domain acts on XBP1 mRNA, performing an unconventional splicing which, after the excision of 26 nucleotides, produces a spliced mRNA (XBP1s) which increases transcription of UPR target genes^1,2^. Mutation of Tyr892, His910 and Asn906 abolished the RNase activity *in vitro*, highlighting their key role in IRE1’s activity^7^. The proposed catalytic mechanism of the RNase site includes His910, Tyr892, Asn906 and Arg905 as residues driving the catalytic reaction, with His910 and Tyr892 (corresponding to His1061 and Tyr1043 in yeast) as the general acid-general base pair and Asn906 and Arg905 responsible for coordination of the scissile phosphate (corresponding to Asn1057 and Arg1056 in yeast)^8^.

The UPR is associated with numerous diseases^9^ and to this end IRE1 has been the focus of several drug discovery projects^10^. Different chemical scaffolds have been identified as IRE1 modulators. The compounds can be categorized as (i) ATP-competitive inhibitors that block the kinase domain and activate RNase^11^, (ii) ATP-competitive inhibitors that affect the kinase domain and inactivate RNase (*i*.*e*., kinase inhibiting RNase attenuators or KIRAs)^11,12^, or (iii) direct IRE1 RNase inhibitors^7^.

The direct RNase inhibitors known to date share a common hydroxy aryl aldehyde (HAA) moiety, which reacts selectively with a specific lysine residue (Lys907) through Schiff base formation in the RNase domain^7^. Crystallographic structures of IRE1 in complex with HAA inhibitors are available (PDB code: 4PL3, 4PL4 and 4PL5). Besides formation of a reversible Schiff base with Lys907, the inhibitors also establish hydrophobic contacts with His910 and Phe889 and a hydrogen bond with Tyr892 in the IRE1 RNase domain. A series of biochemical and computational analysis suggesting increased rate of Schiff base formation and reduced imine bond hydrolysis for this particular Lys907 provided new insights on the site specific reactivity of the HAA compounds^13^.

A detailed understanding of IRE1 activation and connection to RNase activity is not fully available, but several insights have been obtained. Autophosphorylation of the kinase domain is coupled to RNase activity^14,15^. High RNase activity is represented by a dimer in back-to-back conformation^16^, while low RNase activity show protomers in a face-to-face orientation^17^. Lastly, IRE1 endoribonuclease domain activation turned out to be fundamental for the splicing of XBP1^6,18,19^. However, details regarding IRE1 RNase activation and the mechanism of mRNA recognition and cleavage still remain unresolved.

Based on the available studies and crystal structures of murine IRE1α in complex with covalently bound HAA inhibitors we herein investigated the ability of the computational methods of conventional and covalent docking for the identification of novel covalent IRE1 binders in the HAA binding pocket. Docking based methods are commonly used in the development of novel enzyme inhibitors^20^. Conventional docking studies have been extensively applied as a screening strategy for the discovery of covalent ligands^21^. The Schrödinger workflows for covalent docking^22–24^ have been successfully validated on protein targets Cathepsin K, HCV NS3 protease, EGFR, and XPO1, representative of 3 protein families classified as protease, kinase and exportin^22^. However, there are limitations to the applicability of molecular and covalent docking^20,25–27^. In this study, we have assessed the capability of docking-based methods for virtual screening in the RNase active site, and also characterized and compared the druggability parameters for the HAA-based ligand binding sites in the available crystal structures^28^.

## Methods

### Three-dimensional structure of murine Ire1: selection and preparation

At the time of this study, there were only three IRE1 X-ray structures co-crystallized with inhibitors in the RNase domain^7^ present in the Protein Data Bank (PDB)^29^ (Table 1). In addition, data for six other HAAs with reported IC50 values were obtained from the literature (Table S1)^30^. The Schrödinger protein preparation wizard^31^ was used to prepare each crystal structure. Hydrogen atoms were added and possible Metal binding states generated. The protonation and tautomeric states of Asp, Glu, Arg, Lys and His were adjusted to match a pH of 7.4 and possible orientations of Asn and Gln residues were generated. Hydrogen bond sampling with adjustment of active site water molecule orientations was performed using PROPKA at pH 7.4. Water molecules with fewer than two hydrogen bonds to non-waters were deleted. Finally, the protein-ligand complexes were subjected to geometry refinements using the OPLS3 force field^32^ in restrained minimizations. Although the overall resolution of the available crystal structures with PDB code 4PL3,4PL4 and 4PL5 are only 2.9, 3.0 and 3.4 Å, respectively, the ligand binding site has a clear electron density^7^ enabling the docking simulations to be performed without ambiguity.

**Table 1:** Available IRE1 crystallographic structures with HAA inhibitor bound^7^.

PDB code	Ligand name	Resolution (Å)	Organism	Assembly
4PL3	MKC9989	2.9	Mus musculus	dimer
4PL4	OICR464	3.0	Mus musculus	tetramer
4PL5	OICR573	3.4	Mus musculus	tetramer

### Ligand preparation

**Figure 1:**
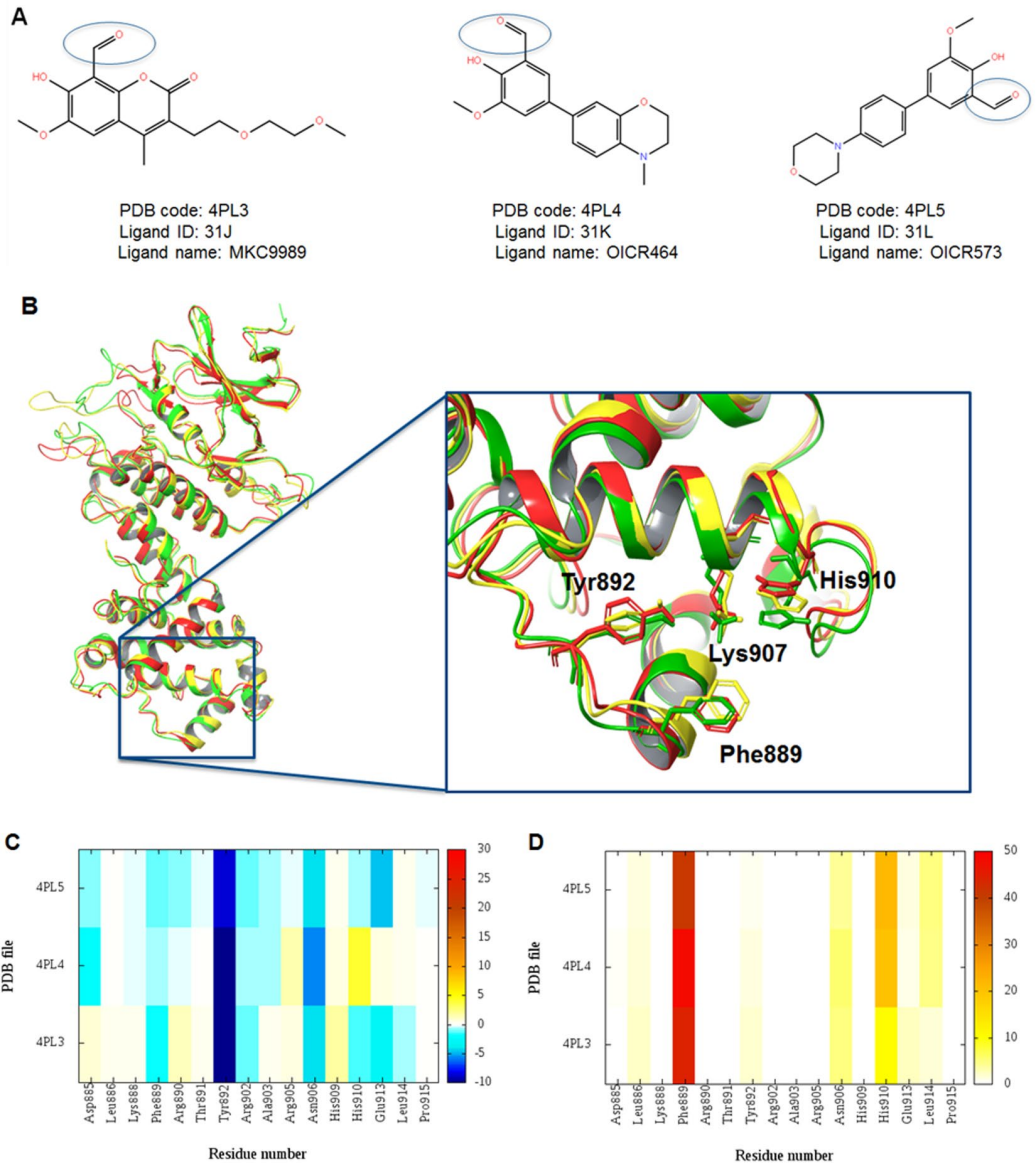
(**A**) Ligands co-crystallized in murine IRE1 RNase active site. Reactive aldehyde groups are highlighted with circles. (**B**) Superposition of the 3D structures of murine IRE1; PDB code 4PL3 (green), 4PL4 (blue), and 4PL5 (red). Key residues involved in binding of HAA are shown in stick model. (**C**,**D**) Per amino-acid interaction energy map for co-crystallized inhibitors in the murine IRE1-HAA binding site. Lys907 is not depicted in the heat maps as it is involved in the covalent bond with the substrates. (**C**) Electrostatic energy values (kcal mol^−1^); (**D**) Hydrophobic score (arbitrary units).

The co-crystallized ligands from Table 1 and the additional six HAA derivatives were extracted and used in docking studies. The ligands are displayed in Fig. 1A and Table S1. The ligands were prepared using LigPrep^33^ in the Schrödinger suite^24^. The OPLS3 force field^32^ was used in all ligand preparation steps. Possible protonation and ionization states were assigned to each ligand using Ionizer at pH 7.4. Possible stereoisomers, tautomeric states and metal binding sites were generated.

### Key Interaction points (KIPs)

Each amino acid residue within 5 Å to the corresponding inhibitors in the IRE1 structure was evaluated for individual electrostatic and hydrophobic contributions to the interaction energy. The electrostatic and hydrophobic contribution were calculated using MOE^34^, with an energy (in kcal/mol) associated with the electrostatic contribution and a score (the higher the better) related to the hydrophobic contribution. Finally, heat maps are used for representing the interaction energy patterns.

### Molecular docking

Docking simulations using the prepared ligand databases were performed using the Glide program^35^ in Schrödinger^24^ with the receptor grid prepared using the OPLS3 force field^32^. The grid center was set to be the centroid of Lys907, and the cubic grid had a side length of 20 Å. No constraints were used in any of the receptor grids. Flexible ligand sampling, using the XP (Extra Precision) docking mode, was considered in the docking procedure. All other parameters were set to defaults for the Glide docking process.

### Covalent docking

Covalent docking simulations were performed using the Covalent Dock Lead Optimization (CovDock-LO) and Virtual Screening (CovDock-VS) Workflows in Schrödinger^22–24^. We were particularly interested in exploring the latter workflow as a possible strategy for structure-based virtual screening (SBVS) for covalent binding drugs in a reasonable computational time-scale.

Performing the covalent docking in Schrödinger requires several key steps.

*Step 1*: Dock the pre-reactive form of the ligand. Before the molecular docking, the reactive amino acid side chain is mutated to alanine. Reactive moieties of the ligand are constrained within 5 Å of the C-beta atom of the reactive residue of the enzyme (*i*.*e*. the side chain carbon of the alanine mutant). In the CovDock-LO workflow conformational sampling and selection of 3 low energy conformations of the ligand are performed. This step is omitted in CovDock-VS. In addition, in the CovDock-LO workflow, poses with a distance <8 Å to the C-beta atom of the reactive residue are retained.

*Step 2*: The mutated amino acid is switched back to the reactive residue. Using a rotamer library, sampling of side-chain conformations is performed, checking if atoms to be involved in the covalent bond are within 5 Å of each other.

*Step 3*: Covalent bond formation.

*Step 4*: Minimization in vacuum of the covalent complexes and clustering of optimized poses. CovDock-VS generate three clusters whereas in CovDock-LO a larger number is generated (~20). In the CovDock-LO workflow, the selected poses are further minimized using the Prime VSGB2.0 energy model^36^.

*Step 5*: Selection and ranking of protein-ligand complexes. In CovDock-LO the selection of the most likely poses (*i*.*e*., binding geometry) and ranking of the compounds is based on an empirical scoring function defined as the averaged Glide score^37,38^ of the binding mode of the pre-reactive species and the approximate Glide score of the ligand in the covalent complex estimation of the bond formation energy. In CovDock-VS, the GlideScore of the binding mode of the pre-reactive species is used to select and rank the protein-ligand complexes.

Earlier benchmark and performance studies of these two programs have confirmed the feasibility of these techniques for covalent structure-based virtual screening (SBVS)^22,23^. CovDock-VS has an easy to implement workflow combining Glide docking and Prime optimization protocols. Importantly, the reactive atoms are automatically detected by specifying the reaction type. This step is crucial as it eliminates manual input errors and is thus adaptable for screening large ligand libraries. Indeed, benchmarking studies showed that the CovDock-VS algorithm was able to screen a large compound collection with a variety of chemical warheads^22^. Using Glide for conventional docking and CovDock for covalent docking, where the latter also uses Glide to estimate the non-covalent interactions, we could compare the impact of modeling the covalent bond formation on the docked pose and scoring of the compounds. To the best of our knowledge, other covalent docking programs show severe limitations when applied to large libraries of compounds^20^. In addition, finding the correct electrophilic warhead of the ligands and dealing with the covalent reaction (*i*.*e*. Schiff-base formation in the current case) is a considerable challenge, and largely limits the applicability of covalent docking procedures^20,26^. Moreover, a comparison between results from non-covalent (Glide XP) and covalent (CovDock) docking methods can provide valuable input towards development a successful docking protocol handling difficult-to-explore systems and automated screening of large databases^26^.

### Molecular Dynamics (MD) simulation

For the highest docking score poses of the IRE1 co-crystallized HAA complex generated with CovDock, MD simulations were performed using the Desmond program^39^. The TIP3P water model^40^ was used to simulate water molecules in a orthorhombic box under periodic boundary conditions, positioned such that the walls were at minimum 10 Å distance from any system atom. Counter ions (*i*.*e*. Na^+^/Cl^−^) were added to balance the system charge. The default Desmond protocol was performed for minimization and relaxation of the IRE1 or IRE1-HAA complexes in the NPT ensemble^39^. Periodic boundary conditions and the OPLS3 force field were applied in the MD simulations^32^. Using Nose-Hoover temperature coupling and isotropic scaling^41^, the temperature and pressure were kept constant at 300 K and 1 atmospheric pressure, respectively. The simulations were run for 5 ns in the NPT ensemble, saving the obtained configurations at 10 ps intervals.

### Druggability assessment of the HAA binding site

The SiteMap module^28^ in Schrödinger^24^ was used to assess the druggability of the HAA binding site, for all three available co-crystallized structures. The volume of the HAA pocket, the enclosure, and the degree of hydrophobicity was used to assess druggability. The sites were scored using Dscore and Site Score values, defined as follow:

1 $$Dscore=0.094\,\sqrt{n}+0.60\,e-0.324\,p$$

2 $$SiteScore=0.0733\sqrt{n}+0.6688\,e-0.20\,p$$

where *n* is the number of site points (capped at 100), *e* is the enclosure score, and *p* is the hydrophilic score. The latter is capped at 1.0 to limit the impact of hydrophilicity in charged and highly polar sites.

Binding sites can be classified based on Dscore, assigning values ≥1.0 as “druggable”, 0.8–1.0 as “intermediate” and those having smaller values than 0.8 as “undruggable”. In general, hydrophobicity is key for a good druggability score, whereas hydrophilic binding sites are difficult to accommodate small organic (“non-polar”) molecules^42^. The SiteMap parameters have been benchmarked on several binding sites^28^, with the hydrophobic and hydrophilic parameters normalized for each site. The size of the site is measured by the number of site points found and the relative openness of the site as measured by exposure and enclosure properties. In the benchmark studies, the average number of site points for a tight binding site was 132. SiteScore is used to identify and compare binding sites, with scores >0.80 found for known binding sites and an average SiteScore for tight binding sites of 1.01. SiteMap also evaluates the size, and the hydrophobic and hydrophilic character of the binding site^28^.

## Results and Discussion

### IRE RNase domain sequence and structural analysis

The primary sequence of the RNase domain of murine IRE1 and human IRE1 (*h*IRE1) are closely related with high sequence identity (>85%) and sequence similarity (>89%). The available structural data were also examined (Figs S1 and S2). Based on the 3D superposition of the RNase domain of known IRE1α structures, the Cα RMSD comparison displays highly similar conformations among all structures (Figs S1 and S2). The 3D structures of the RNase domain of murine IRE1 show less than 1 Å displacement to those of human IRE1. In addition, crucial residues for IRE1-HAA interaction are conserved, both in terms of sequence and 3D conformation (Figs S1, S2 and S3).

Also the crystal structures with HAA derivatives bound were aligned (Fig. 1B), and display highly similar conformations (Cα RMSDs ~1 Å) (Fig. S2). For this reason, only one crystal structure, PDB code: 4PL3 with inhibitor MKC9989 co-crystallized, was used as a representative structure in the molecular docking studies.

### Dissecting IRE1 RNase-HAA interactions

Different structural information for the IRE1 RNase inhibitor complexes were investigated as a means to characterize crucial interactions in the binding site. To investigate the HAA-IRE1 recognition mechanism in a quantitative manner, we calculated the individual electrostatic and hydrophobic contributions to the interaction energy of each amino-acid residue required in the binding with the co-crystallized inhibitors. The calculated per residue electrostatic and hydrophobic energy interaction contributions values are depicted as heat maps shown in Fig. 1C,D.

The electrostatic KIPs of the co-crystallized structures (Fig. 1C) display at least two residues with favourable interaction with the co-crystallized inhibitors (coloured blue), namely Tyr892 and Asn906. For co-crystallized ligands MKC9989 and OICR573 favorable electrostatic interactions are also established with Glu913 (Fig. S4).

On the other hand, the hydrophobic KIPs (Fig. 1B,D) reveal mainly Phe889 and His910, alongside Asn906 which also has electrostatic interaction, as involved in hydrophobic contacts with the co-crystallized compounds. In addition, analysis of the reported crystal structures demonstrates that lysine 907 is involved in a Schiff-base arrangement (Fig. S4), consistent with available data^7^. To summarize, the KIPs highlight that the co-crystallized compounds share a common binding mode in the murine IRE1 RNase domain.

In order to decipher the mode of action of the HAA inhibitors, we investigated through visual inspection, the proximity of the ligand binding site to the catalytic residues involved in site-specific cleavage of XBP1 mRNA. The experimental mutation data available on His910, Tyr892 and Asn906 greatly impact RNase activity^8^. These three amino-acids are conserved throughout the species in terms of both sequence and 3D structure (Figs S1, S2 and S3). For Lys907 and Phe889 the experimental data is inconclusive. Mutation of these residues abolished RNase activity using a single hairpin RNA substrate, while detectable RNase activity could be seen when using a double hairpin RNA substrate^7^. As clearly evidenced in Fig. 1B, the MKC9989 compound could disfavour IRE1 RNase activity by interfering with the cleavage of XBP1 mRNA by blocking access to the RNase active site.

### Conventional docking analysis

**Figure 2:**
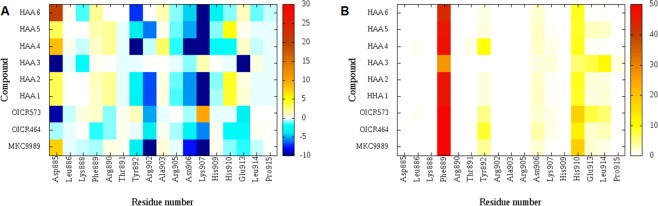
Per amino-acid interaction energy map for a dataset of hydroxy aryl aldehyde (HAA) class of IRE1 endoribonuclease inhibitors docked inside the binding site of MKC9989 using conventional docking studies (PDB code: 4PL3). (**A**) Electrostatic energy values (kcal mol^−1^); (**B**) Hydrophobic score (arbitrary units).

As a next step we decided to perform a conventional (non-covalent) docking study to investigate the suitability of this pocket as a candidate target site for virtual screening. A dataset of HAA types of IRE1 endoribonuclease inhibitors^30^ (Table S1) was docked into the available binding site of MKC9989 (PDB code: 4PL3). The results highlight that the major electrostatic and hydrophobic interaction are conserved through the HAA class of IRE1 endoribonuclease inhibitors. The per-residue electrostatic KIPs (Fig. 2A) indicates at least three fundamental groups with favourable electrostatic interaction with the dataset of compounds (coloured in blue), namely Arg902, Asn906 and Lys907. Also, the map of the per-residue hydrophobic interactions (Fig. 2B) highlights at least two residues, Phe889 and His910, involved in hydrophobic contacts.

In addition, the conventional non-covalent docking of the pre-reactive species underlines the importance of the Lys907 residue. The reactive aldehyde group in the ligand dataset is in close proximity to the reactive Lys907 (O^…^N distance ~3 Å) (Fig. S5 and Table S2). Understandably, there are few exceptions (Fig. S6). The aldehyde moiety in OICR464 is involved in favourable electrostatic interaction with Asn906, explaining the large distance of the carbonyl group from Lys907 (~9 Å) (Figs S6, S7 and S8). For OICR573 the best-ranked pose maximizes the electrostatic and hydrophobic interactions among the amino acids surrounding the HAA binding pocket, explaining the 4.1 Å distance between the two reactive groups (Figs S6, S7 and S8). For HAA 3 (Table S1) the third-ranked pose allowed proximal vicinity between the Lys907 and the reactive part of the ligand. This is not true for OICR464 and OICR573 where the additional poses generated by Glide were not able to accommodate in close proximity the aldehyde moiety with the carbonyl group from Lys907.

These three exceptions (OICR464, OICR573 and HAA3) also explain the differences in the KIPs displayed in Fig. 4, in that those compounds establish favourable electrostatic interaction with Asp885 whereas for the rest of the dataset this is a repulsive interaction (Fig. S8).

**Figure 3:**
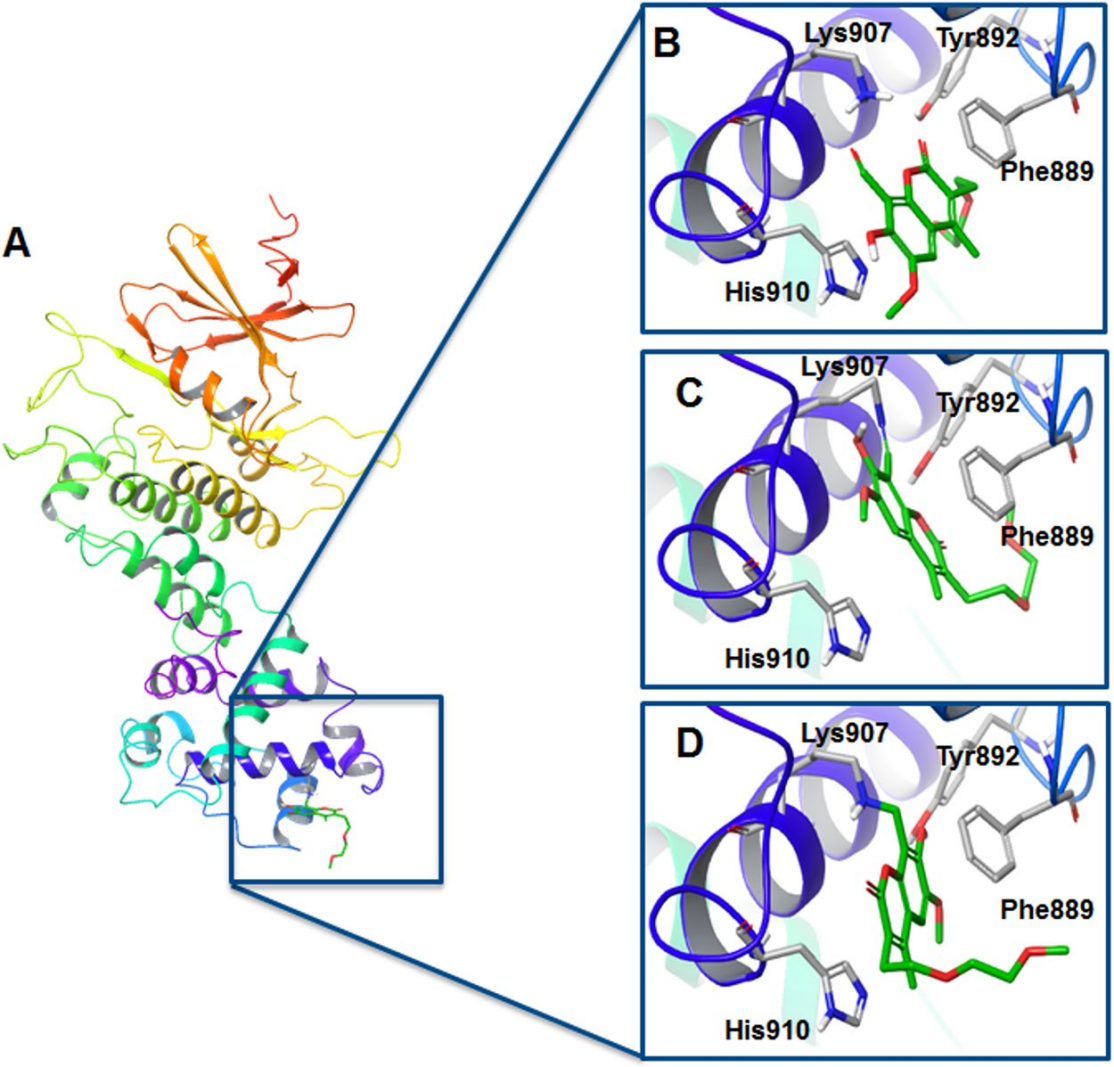
(**A**) Crystal structure of murine IRE1 with MKC9989 bound to the binding pocket of the RNase active site (PDB 4PL3). Comparison of (**B**) conventional docked pose, (**C**) Covalent Docking pose using VS workflow, and (**D**) crystal pose of MKC9989.

Despite the docking poses for the most parts showing close proximity between reactive groups (Table S2), the predicted poses differ from the crystal pose as illustrated in Fig. 3. For the predicted pose of MKC9899, the [2-(2-methoxyethoxy)ethyl] substitution in position 3 lies inside the binding cavity (Fig. 3B), while in the co-crystallized pose the substituent points away from the binding pocket (Fig. 3D). The docked pose is furthermore rotated 180° compared to crystallographic pose. This is an understandable result since neglecting the surrounding water in the docking procedure allows the [2-(2-methoxyethoxy)ethyl] substitution to establish favourable interaction with the target, while under physiologic conditions (*i*.*e*. with solvent molecules present), the substituent will be more likely to interact with water.

In addition, the ligands score poorly inside the active site (Table S1). The docking scores of the unbound pre-reactive species confirm the covalent bond as necessary for strong interaction with the target. The poor docking score of the pre-reactive ligands should discard users from using this pocket site as a candidate target for conventional non-covalent virtual screening studies, as ranking ligands would be highly problematic. This is a serious limitation and a fundamental pre-requisite for a successful virtual screening campaign^43^.

### Druggability of the HAA binding pocket

The druggability of the HAA binding pockets obtained from the available co-crystallized structures (PDB codes 4PL3, 4PL4 and 4PL5) were analysed. We evaluated the HAA binding site region within 6 Å from the co-crystallized ligands. The druggable sites were scored using Dscore values obtained from the SiteMap module in Schrödinger^24^ (Table 2). Different binding sites can be classified based on Dscore, assigning values ≥1.0 as “druggable”, 0.8–1.0 as “intermediate” and those having smaller values than 0.8 as “undruggable”^28^.

**Table 2:** Different properties of the HAA binding pocket in the IRE1 RNase domain of the crystal structures.

PDB code	Ligand name	DScore^28^	SiteScore^28^	Size^28^	hydrophilic^28^	hydrophobic^28^
4PL3	MKC9989	0.570	0.618	32	0.940	0.253
4PL4	OICR464	0.462	0.585	30	1.216	0.042
4PL5	OICR573	0.366	0.461	15	1.022	0.250

For all the systems, DScore categorize this pocket as “undruggable” for non-covalent binders. The main characterics of undruggable sites are that they are small, strongly hydrophilic and with very little hydrophobic character. In addition, we used SiteScore to validate the druggability classification as obtained from the Dscore analysis. The SiteScore criterion of at least 0.80 discriminates a putative drug binding site from an undruggable one. All the values are below 0.80, confirming again the information received from Dscore. Again, the binding site analysis clearly reveal lack of an evident druggable pocket. Hence, this binding site may not be suitable for conventional non-covalent virtual screening studies. This is also in line with the very low docking scores obtained for all the nine compounds studies (Table S1).

### Covalent docking analysis

**Figure 4:**
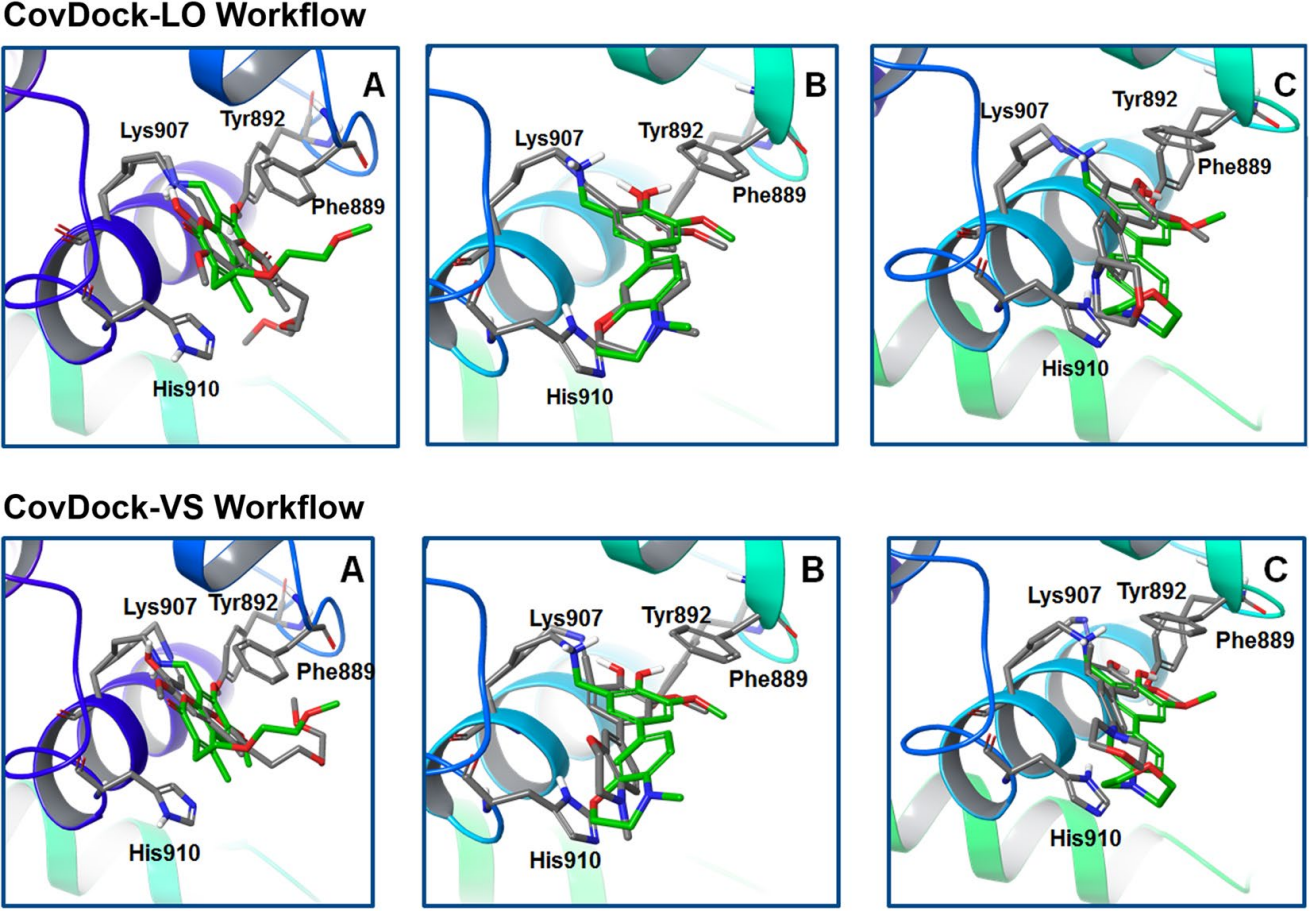
Superposition of the best-scoring docked poses from covalent docking (grey) onto the native crystal one (green). Lys907 and key residues in the binding site are highlighted and all other residues are hidden for clarity (**A**) MKC9989 (PDB code: 4PL3) (**B**) OICR464 (PDB code: 4PL4) (**C**) OICR573 (PDB code: 4PL5).

Next, we evaluated the performance of the CovDock-LO and CovDock-VS workflows^22,23^ available in the Schrödingher package^24^, in predicting the ligand binding pose in the IRE1 RNase site (Fig. 4).

The superposition of each best-scoring docked poses onto the related crystal structure is shown in Fig. 4. Except for the pose generated for OICR464 using the CovDock-LO approach, both workflows were unable to correctly reproduce the crystallographic ligand binding modes. The RMSD values were >2 Å, *i*.*e*. beyond the threshold to consider the docking as successful (Table S3). Visual inspection and RMSD analysis of all the poses generated by the program were performed as well, without displaying any successful results (*i*.*e*. RMSD <2 Å).

An additional minimization and relaxation using 5 ns explicit solvent MD simulations were performed on each best-scoring docked pose. The final structures from the trajectories were compared with the crystal structures. The results demonstrate that short MD simulations are still not enough to correctly reproduce the crystallographic IRE1-HAA binding mode (Fig. S9). In addition, we investigated the ability of the workflow to correctly rank ligands according to experimental studies available^7^. As clearly evidenced in Table 3, the program used is not able to rank the three co-crystallized HAA inhibitors in agreement with the binding assay data available in the literature^7^. This is not unexpected since covalent docking neglects to explicitly explore the reactivity of the given covalent inhibitors.

**Table 3:** CovDock Glidescore and experimentally measured activity obtained using Micro-scale thermophoresis.

Structure	Ligand	Averaged Glide score^a^ using CovDock- LO Workflow (default mode)	GlideScore^b^ using CovDock- VS Workflow	K_d_ (μM)^c^
4PL3	MKC9989	−4.978	−4.710	0.84
4PL4	OICR464	−4.130	−5.072	15.9
4PL5	OICR573	−5.269	−5.614	35.2
4PL3	HAA1	−3.392	−3.995	N.A.
4PL3	HAA2	−3.673	−4.672	N.A.
4PL3	HAA3	−4.238	−4.969	N.A.
4PL3	HAA4	−3.052	−4.124	N.A.
4PL3	HAA5	−3.043	−3.314	N.A.
4PL3	HAA6	−4.154	−5.158	N.A.

N.A. = Not available. ^a^Averaged score of the binding mode of the pre-reactive species and the approximate score of the ligand in the covalent complex. ^b^GlideScore of the binding mode of the pre-reactive species. ^c^K_d_ values obtained in a direct binding assay^7^.

## Conclusions and Perspective

We have investigated a series of covalent HAA inhibitors of the IRE1 RNase domain. Using *in silico* structure-based approaches, we analyzed and compared the most crucial interactions of the inhibitors in the crystal structures. The reported HAA inhibitors co-crystallized in murine IRE1 highlights favorable electrostatic interaction with Tyr892, hydrophobic contacts with Phe889 and His910 and a Schiff-base arrangement with Lys907.

In addition, the close proximity between the co-crystallized HAA inhibitors and IRE1 residues involved in the cleavage of mRNA XBP1 transcription factor allowed us conclude that the HAA inhibitors might interfere with XBP1 mRNA cleavage by sterically blocking the space required for its recognition.

At a later stage, we focused on the limitations and challenges in using molecular docking approaches to identify new IRE1 RNase modulators. In agreement with experimental results, the conventional docking analysis highlights the importance of Lys907, Tyr892, Phe889 and His910 for the correct accommodation of these HAA inhibitors in the pocket site. In addition, for almost all the inhibitors analyzed, the docked pose of the pre-reactive species is predisposed to form a covalent bond described by the close proximity between the reactive aldehyde group in the ligand dataset and the side chain nitrogen of the reactive Lys907. However, the estimated docking scores using conventional docking were very low. This is a serious limitation in the performance of non-covalent screening towards the HAA binding pocket. The low docking scores confirm the covalent bond formation as absolutely necessary for inhibition, and prevents from ranking compounds in conventional virtual screening studies.

To address this issue, covalent docking analysis of the co-crystallized ligands within the HAA binding pocket were performed. Reproducing the covalently bound conformations of the co-crystallized ligands by covalent docking turned out to be challenging. In addition, the docking scores generated by the CovDock-LO and CovDock-VS modules were unable to accurately reproduce and correctly rank the experimental binding data of the three co-crystallized structures. Although the covalent docking methodologies have been successful in screening large libraries of chemical probes^44^ and enzyme inhibitors^45,46^, at the same time this screening approach has to be evaluated on a case-to-case basis. The success depends on various factors such as the physicochemical properties of the binding site, the reaction mechanism of covalent bond formation, and the extent of non-covalent interactions involved in binding to the target^26^. In this case, the IRE1-HAA non-covalent interactions are weak, and the binding is mostly governed by the covalent bond formation. Since bond formation is not considered explicitly in the scoring function^23^, this results in low docking scores and thereby making the overall protocol unsuccessful for a VS campaign. The data points to limitations of covalent docking use for virtual screening of the IRE1 HAA pocket.

From this perspective, hybrid approaches that combine quantum mechanical (QM) and molecular mechanical (MM) methods could be a possible alternative solution, although this is not appropriate for screening of large databases. QM/MM methods are well established, and provide more accurate estimates of reaction mechanisms, activation energies and covalent binding energies for the rational design of covalent inhibitors^47^. Using available crystal structures and computing the reaction course backward to the corresponding noncovalent IRE1··· HAA complex, novel ligands could be designed in order to increase the inhibition potency of already known inhibitors. The newly designed compounds can be subsequently tested by further QM/MM computations and obtained trends in reactivity validated using experimental studies.

## Supplementary information

Supplementary Material

## Data Availability

The datasets generated or analysed during the current study are available from the corresponding author on reasonable request.
