# Supplementary material for: Merits and pitfalls of conventional and covalent docking in identifying new hydroxyl aryl aldehyde like compounds as human IRE1 inhibitors
Source: Sci Rep. 2019 Mar 4;9:3407. doi: 10.1038/s41598-019-39939-z (PMC6399222; doi:10.1038/s41598-019-39939-z)
Supplement: Supplementary file 1 — Supplementary Material [file 41598_2019_39939_MOESM1_ESM.pdf]

Merits and pitfalls of conventional and covalent docking in identifying new hydroxyl aryl aldehyde like compounds as human IRE1 inhibitors

Antonio Carlesso[1], Chetan Chintha[2], Adrienne M. Gorman[2], Afshin Samali[2] and Leif A. Eriksson[1*]

[1]Department of Chemistry and Molecular Biology, University of Gothenburg, 405 30 Göteborg, Sweden

[2]Apoptosis Research Centre, National University of Ireland Galway, Galway, Ireland.

[*]*Correspondence*
Leif Eriksson, Department of Chemistry and Molecular Biology,
University of Gothenburg, 405 30 Göteborg, Sweden.
Email: leif.eriksson@chem.gu.se

SUPPLEMENTARY INFORMATION

Page

Table S1. Structures and docking score of hydroxy aryl aldehydes (HAA) studied herein. **S3-S4**

Figure S1. (A) RNase domain superposition of all available IRE1 3D structures from yeast, murine and human, (B) Key Residues involved in the binding with HAA derivatives are represented using stick model. **S5**

Figure S2. Root-mean-square deviation (RMSD) matrix values in Å of the position of the Cα atoms for each pair of IRE1 RNase domains. Each row corresponds to a given crystal structure with its PDB ID. The RMSD is represented by a colorimetric scale, going from blue (low) to red (high) RMSD values. The box marks the three structures with co-crystallized ligands. Resolution of the crystal structures is shown as separate column next to the PDB ID. **S6**

Figure S3. Multiple sequence alignment of the IRE1 cytosolic domain in 8 different organisms using Clustal[1]. **S7-S8**

Figure S4. Schematic representation of IRE1 - ligand interactions. **S9-S10**

Figure S5. N-O distance between reactive moieties of the MKC9989 (aldehyde group) and reactive residue of IRE1 (Lysine 907). **S11**

Figure S6. N-O distance between reactive moieties (Lysine 907 of IRE1 and aldehyde group of ligand) of (A) OICR464, (B) OICR573, (C) HAA3. **S12-S13**

Table S2. N-O distance between reactive moieties of the HAA ligands (aldehyde group) and reactive residue of IRE1 (Lysine 907). **S14**

Figure S7. Predicted pose in the binding pocket of the murine RNase active site (PDB 4PL3) of (A) HAA1, (B) HAA2, (C) OICR464, (D) OICR573, (E) HAA3, (F) HAA4, (G) HAA5, (H) HAA6. **S15-S16**

Figure S8. Schematic representations of the predicted pose in the binding pocket of the murine RNase active site (PDB 4PL3) of (A) MKC889, (B) OICR464, (C) OICR573, (D) HAA1, (E) HAA2, (F) HAA3, (G) HAA4, (H) HAA5, and (I) HAA6. **S17-S21**

Table S3. Self-docking pose prediction for available crystal structure complexes. **S22**

Figure S9. Superposition between the experimental crystallographic complex and the last frame of the MD simulations for docked poses generated using (A) CovDock LO and (B) CovDock VS for MKC9989 bound (PDB 4PL3); (C) CovDock LO and (D) CovDock VS for OICR464 (PDB 4PL4); (E) CovDock LO and (F) CovDock VS for OICR573 (PDB 4LP5). **S23-S25**

Bibliography **S26**

Table S1. Structures and docking score of hydroxy aryl aldehydes (HAA) studied herein.

Structure	Ligand Name	Docking Score	IC50 (μM)	Reference
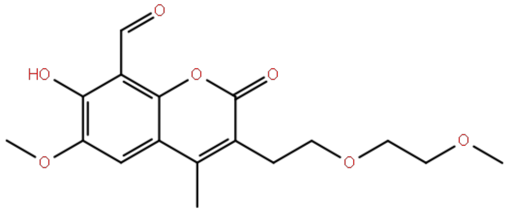	MKC9989	-4.493	0.23 ± 0.03	[2]
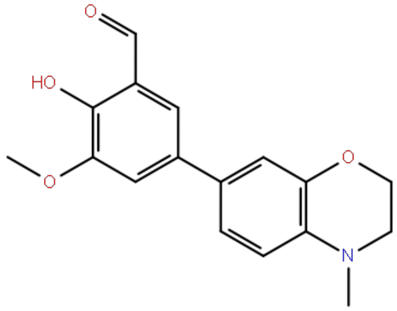	OICR464	-3.257	2.3 ± 0.69	[2]
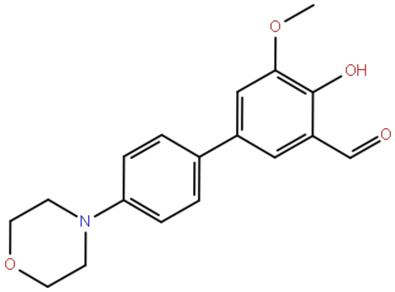	OICR573	-3.807	5.4 ± 1.9	[2]
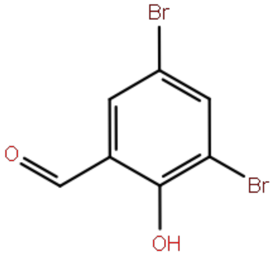	HAA 1	-2.712	0.54	[3]
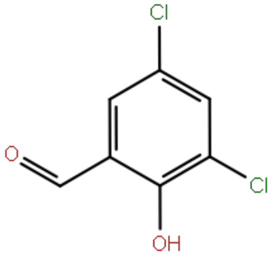	HAA 2	-2.749	1.77	[3]

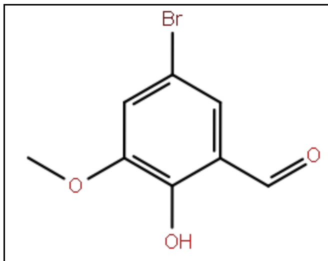	HAA 3	-2.481	0.64	[3]
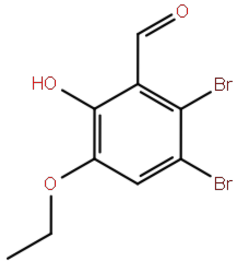	HAA 4	-2.770	0.12	[3]
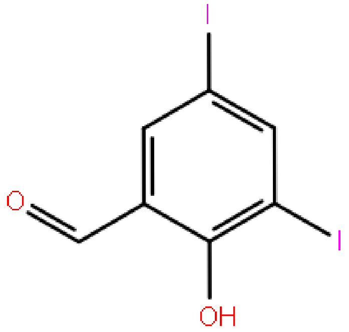	HAA 5	-2.485	0.42	[3]
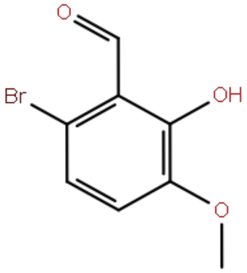	HAA 6	-2.939	0.41	[3]

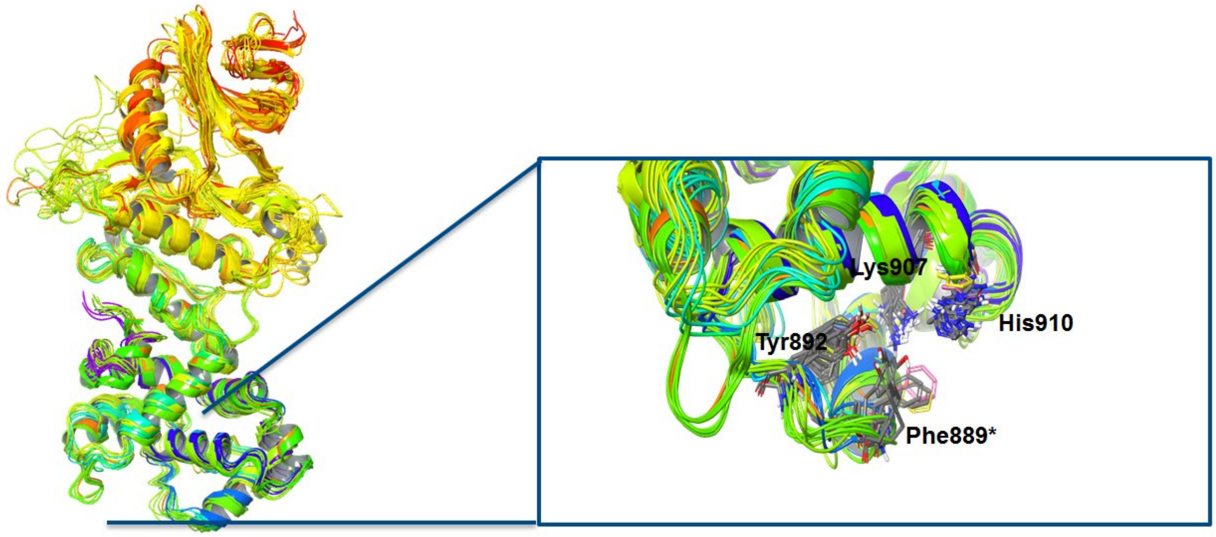

Figure S1. RNase domain superposition of all available IRE1 3D structures from yeast, murine and human. Key residues involved in the binding with HAA derivatives are represented using stick model. *Phe899 is mutated in Tyr in yeast.

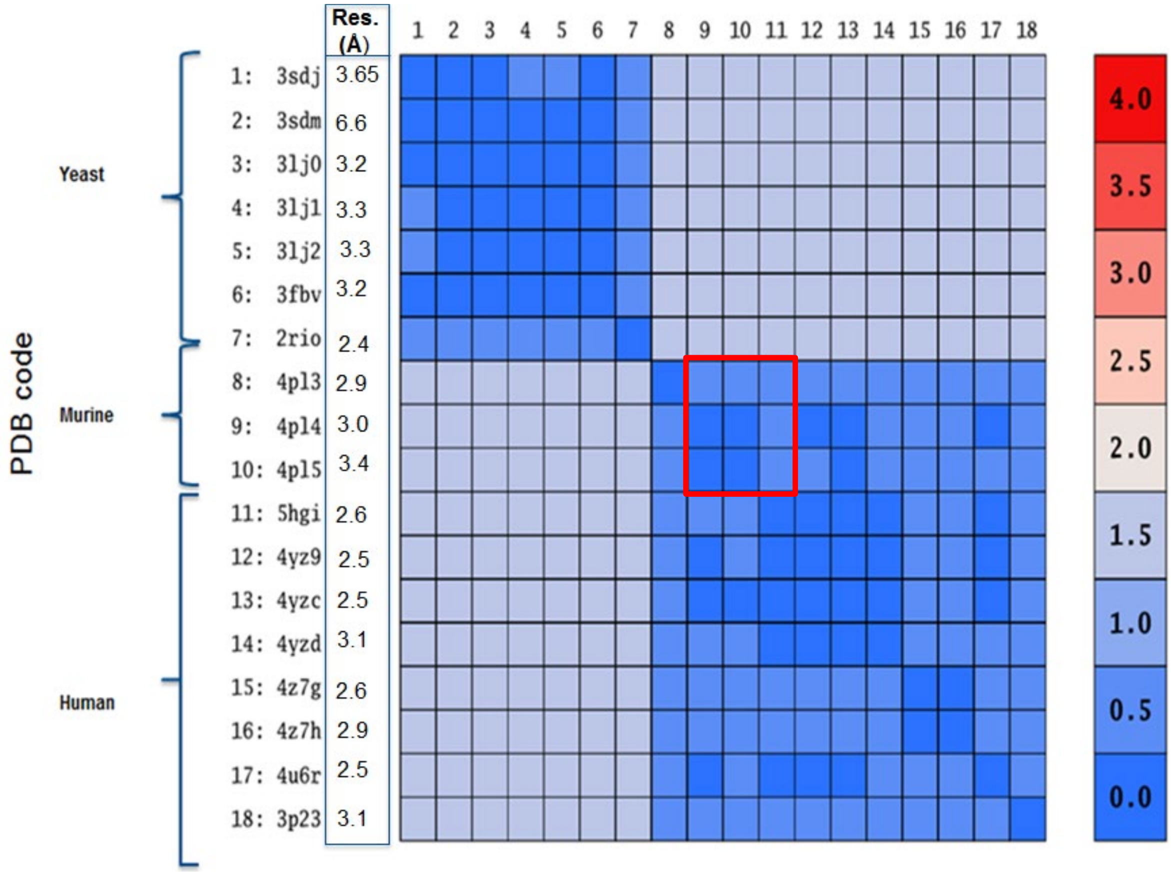

Figure S2. Root-mean-square deviation (RMSD) matrix values in Å of the position of the Cα atoms for each pair of IRE1 RNase domains. Each row corresponds to a given crystal structure with its PDB code. The RMSD is represented by a colorimetric scale, going from blue (low) to red (high) RMSD values. The box marks the three structures with co-crystallized ligands. Resolution of the crystal structures is shown as separate column next to the PDB code.

```
CLUSTAL O(1.2.4) multiple sequence alignment


Aotus nancymaae     ------------------------GSSPTLEQDDGDEETSMVMVGKISFCPKDVLGHGAE      36
Pongo abelii        ------------------------GSSPSLEQDDGDEETSMVIVGKISFCPKDVLGHGAE      36
Homo sapiens        MGHNHRHKHHHHHHHHENLYFQGPGSSPSLEQDDGDEETSVVIVGKISFCPKDVLGHGAE      60
Papio anubis        ------------------------GSSPSLEQDDGDEETSMVIVGKISFCPKDVLGHGAE      36
Piliocolobus t.     ------------------------GGSPSLEQDDGDEETSMVIVGKISFCPKDVLGHGAE      36
Ictidomys t.        -----------NHSLHSGSSVSKAGAGPFLEQDEEDEETSMVIVGKISFCPKDVLGHGAE      49
Felis catus         -----------NHSLHSSGSASRAGASPFLDQDDEDEETSMVIVGKISFCPKDVLGHGAE      49
Canis lupus f.      ---------------------------PFLEQDDEDEETSMVIVGKISFCPKDVLGHGAE      33
                                               * *:**: *****:*:*****************

Aotus nancymaae     GTIVYRGMFDNRDVAVKRILPECFSFADREVQLLRESDEHPNVIRYFCTEKDRQFQYIAI      96
Pongo abelii        GTIVYRGMFDNRDVAVKRILPECFSFADREVQLLRESDEHPNVIRYFCTEKDRQFQYIAI      96
Homo sapiens        GTIVYRGMFDNRDVAVKRILPECFSFADREVQLLRESDEHPNVIRYFCTEKDRQFQYIAI      120
Papio anubis        GTIVYRGMFDNRDVAVKRILPECFSFADREVQLLRESDEHPNVIRYFCTEKDRQFQYIAI      96
Piliocolobus t.     GTIVYRGMFDNRDVAVKRILPECFSFADREVQLLRESDEHPNVIRYFCTEKDRQFQYIAI      96
Ictidomys t.        GTIVYRGMFDNRDVAVKRILPECFSFADREVQLLRESDEHPNVIRYFCTERDRQFQYIAI      109
Felis catus         GTIVYRGMFDNRDVAVKRILPECFSFADREVQLLRESDEHPNVIRYFCTERDRQFQYIAI      109
Canis lupus f.      GTIVYRGMFDNRDVAVKRILPECFSFADREVQLLRESDEHPNVIRYFCTERDRQFQYIAI      93
                    **************************************************:*********

Aotus nancymaae     ELCAATLQEYVEQKDFAHLGLEPITLLQQTTSGLAHLHSLNIVHRDLKPHNILISMPNAH      156
Pongo abelii        ELCAATLQEYVEQKDFAHLGLEPITLLQQTTSGLAHLHSLNIVHRDLKPHNILISMPNAH      156
Homo sapiens        ELCAATLQEYVEQKDFAHLGLEPITLLQQTTSGLAHLHSLNIVHRDLKPHNILISMPNAH      180
Papio anubis        ELCAATLQEYVEQKDFAHLGLEPITLLQQTTSGLAHLHSLNIVHRDLKPHNILISMPNAH      156
Piliocolobus t.     ELCAATLQEYVEQKDFAHLGLEPITLLQQTTSGLAHLHSLNIVHRDLKPHNILISMPNAH      156
Ictidomys t.        ELCAATLQEYVEQKDFAHLGLEPITLLQQTTSGLAHLHSLNIVHRDLKPHNILLSMPNAH      169
Felis catus         ELCAATLQEYVEQKDFAHLGLEPITLLQQTTSGLAHLHSLNIVHRDLKPHNILLSMPNAH      169
Canis lupus f.      ELCAATLQEYVEQKDFAHLGLEPITLLQQTTSGLAHLHSLNIVHRDLKPHNILLSMPNAH      153
                    *****************************************************:******

Aotus nancymaae     GKIKAMISDFGLCKKLAVGRHSFSRRSGVPGTEGWIAPEMLSEDCKENPTYTVDIFSAGC      216
Pongo abelii        GKIKAMISDFGLCKKLAVGRHSFSRRSGVPGTEGWIAPEMLSEDCKENPTYTVDIFSAGC      216
Homo sapiens        GKIKAMISDFGLCKKLAVGRHSFSRRSGVPGTEGWIAPEMLSEDCKENPTYTVDIFSAGC      240
Papio anubis        GKIKAMISDFGLCKKLAAGRHSFSRRSGVPGTEGWIAPEMLSEDCKENPTYTVDIFSAGC      216
Piliocolobus t.     GKIKAMISDFGLCKKLAAGRHSFSRRSGVPGTEGWIAPEMLSEDCKENPTYTVDIFSAGC      216
Ictidomys t.        GRIKAMISDFGLCKKLAVGRHSFSRRSGVPGTEGWIAPEMLSEDCKENPTYTVDIFSAGC      229
Felis catus         GRIKAMISDFGLCKKLAVGRHSFSRRSGVPGTEGWIAPEMLSEDCKDNPTYTVDIFSAGC      229
Canis lupus f.      GRIKAMISDFGLCKKLAVGRHSFSRRSGVPGTEGWIAPEMLSEDCKDNPTYTVDIFSAGC      213
                    *:***************.****************************:*************

Aotus nancymaae     VFYYVISEGSHPFGKSLQRQANILLGACSLDSLHPEKHKDVIARELIEKMIAMDPQKRPS      276
Pongo abelii        VFYYVISEGSHPFGKSLQRQANILLGASSLDCLHPEKHEDVIARELIEKMIAMDPQKRPS      276
Homo sapiens        VFYYVISEGSHPFGKSLQRQANILLGACSLDCLHPEKHEDVIARELIEKMIAMDPQKRPS      300
Papio anubis        VFYYVISEGSHPFGKSLQRQANILLGACSLECLHPEKHEDVIARELIEKMIAMDPQKRPS      276
Piliocolobus t.     VFYYVISEGSHPFGKSLQRQANILLGACSLECLHPEKHEDVIARELIEKMIAMDPQKRPS      276
Ictidomys t.        VFYYVISEGSHPFGKSLQRQANILLGAYSLECFHPEKHEDVIARELIEKMIAMDPQQRPS      289
Felis catus         VFYYVISEGSHPFGKSLQRQANILLGAYSLDCLHPEKHEDVIARELIEKMIAMDPQKRPS      289
Canis lupus f.      VFYYVISEGSHPFGKSLQRQANILLGAYNLDCLHPEKHEDVIARELIEKMIAMDPQKRPS      273
                    *************************** .*:.:*****:*****************:***

Aotus nancymaae     AKHVLKHPFFWSLEKQLQFFQDVSDRIEKESLDGPIVKQLERGGRAVVKMDWRENITVPL      336
Pongo abelii        AKHVLKHPFFWSLEKQLQFFQDVSDRIEKESLDGLIVKQLERGGRAVVKMDWRENITVPL      336
Homo sapiens        AKHVLKHPFFWSLEKQLQFFQDVSDRIEKESLDGPIVKQLERGGRAVVKMDWRENITVPL      360
Papio anubis        AKHVLKHPFFWSLEKQLQFFQDVSDRIEKESLDGPIVKQLERGGRAVVKMDWRENITVPL      336
Piliocolobus t.     AKHVLKHPFFWSLEKQLQFFQDVSDRIEKESLDGPIVKQLERGGRAVVKMDWRENITVPL      336
Ictidomys t.        AKHVLKHPFFWSLEKQLQFFQDVSDRIEKESLDGPIVRQLERGGRAVVKMDWRENITVPL      349
Felis catus         AKHVLKHPFFWSLEKQLQFFQDVSDRIEKESLDGPIVKQLERGGRAVVKTDWRENITVPL      349
Canis lupus f.      AKHVLKHPFFWSLEKQLQFFQDVSDRIEKESLDGPIVKQLERGGRSVVKMDWRENITVPL      333
                    ********************************** **:*******:*** **********

Aotus nancymaae     QTDLRKFRTYKGGSVRDLLRAMFNKKHHYRELPPEVRETLGSLPDDFVCYFTSRFPHLLA      396
Pongo abelii        QTDLRKFRTYKGGSVRDLLRAMFNKKHHYRELPAEVRETLGSLPDDFVCYFTSRFPHLLA      396
Homo sapiens        QTDLRKFRTYKGGSVRDLLRAMFNKKHHYRELPAEVRETLGTLPDDFVCYFTSRFPHLLA      420
Papio anubis        QTDLRKFRTYKGGSVRDLLRAMFNKKHHYRELPAEVRETLGSLPDDFVCYFTSRFPHLLA      396
Piliocolobus t.     QTDLRKFRTYKGGSVRDLLRAMFNKKHHYRELPAEVRETLGSLPDDFVCYFTSRFPHLLA      396
Ictidomys t.        QTDLRKFRTYKGGSVRDLLRAMFNKKHHYRELPVEVRETLGSLPDDFVRYFTSRFPHLLS      409
Felis catus         QTDLRKFRTYKGGSVRDLLRAMFNKKHHYRELPAEVRETLGSLPDDFVRYFTSRFPHLLP      409
Canis lupus f.      QTDLRKFRTYKGGSVRDLLRAMFNKKHHYRELPAEVRETLGSLPDDFVRYFTSRFPHLLS      393
```

```
                    ********************************* *******:****** **********

Aotus nancymaae     HTYRAMELCSHERLFQPYYFHEPLQPQPPVTPGAL  431
Pongo abelii        HTYQAMELCSHERLFQPYYFHEPPEPQPPVTPDAL  431
Homo sapiens        HTYRAMELCSHERLFQPYYFHEPPEPQPPVTPDAL  455
Papio anubis        HTYRAMELCSHERLFQPYYFHRPPEPQPPVTPDAL  431
Piliocolobus t.     HTYRAMELCSHERLFQPYYFHRPPEPQPPVTPDAL  431
Ictidomys t.        HTYRAMELCSHERLFQPYYFHEPLEPQPPVTPDAL  444
Felis catus         HTYRAMEPCSHERLFQPYYFHEPPELRPPVTPDAL  444
Canis lupus f.      HTYRAMEPCSHERLFQPYYFHEPPDTRPPVTPDTL  428
                    ***:*** *************.* : :*****.:*
```

Figure S3. A multiple sequence alignment of the IRE1 cytosolic domain in 8 different organisms using Clustal[1]. Residues are coloured as follow: AVFPMILW are shown in red, DE are blue, RHK are magenta, STYHCNGQ are green. IRE1 Catalytic residues involved in the cleavage of m-RNA XBP1-transciption factor [4] and amino-acids involved in binding with HAA co-crystallized are highlighted using blue boxes.

A

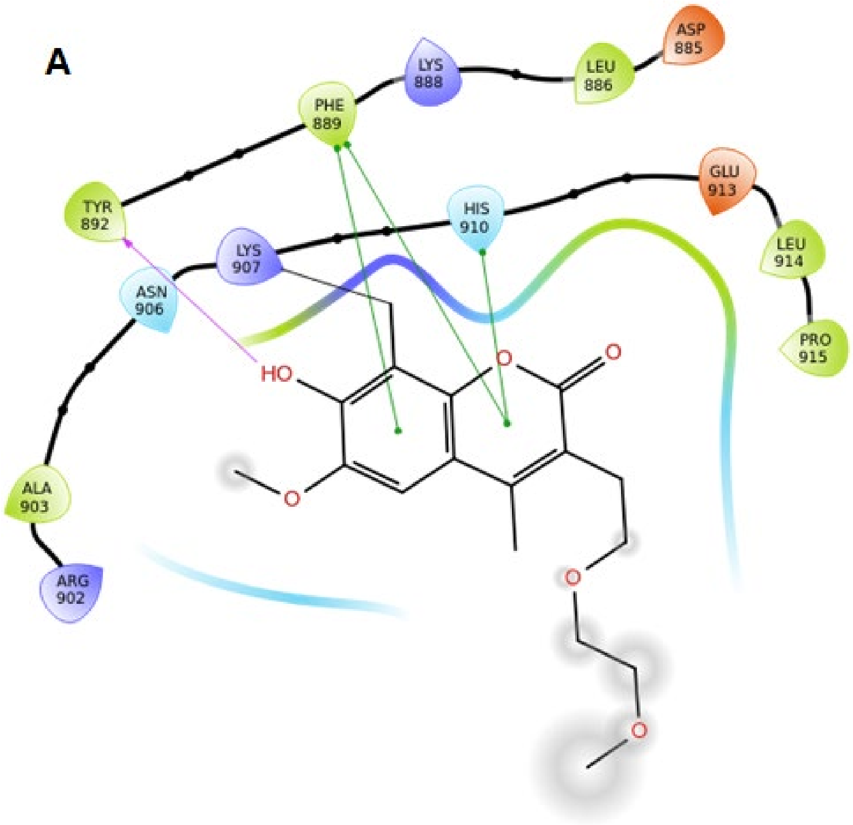

ASP 885
LEU 886
LYS 888
PHE 889
TYR 892
GLU 913
HIS 910
LYS 907
ASN 906
LEU 914
PRO 915
ALA 903
ARG 902
HO
O
O
O
O
O

B

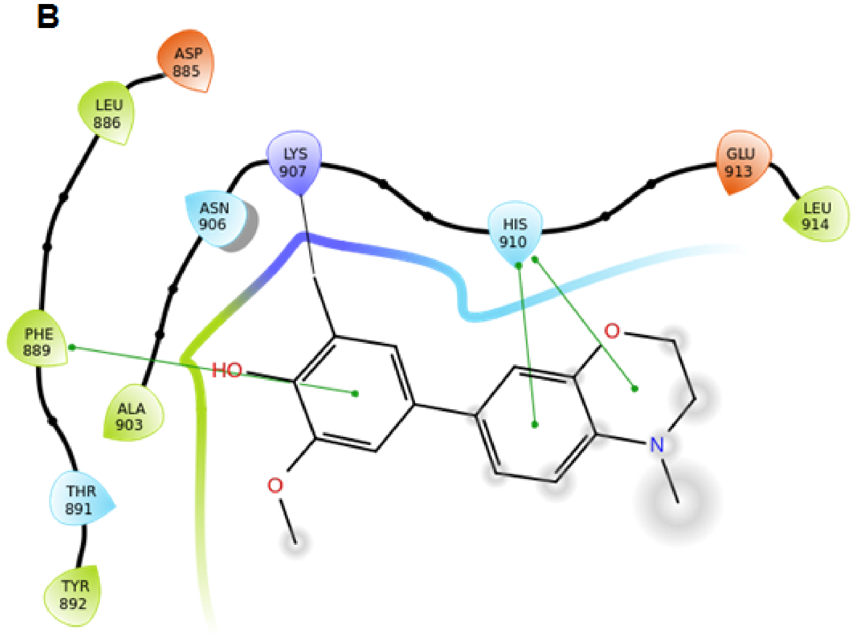

ASP 885
LEU 886
LYS 907
ASN 906
GLU 913
HIS 910
LEU 914
PHE 889
ALA 903
THR 891
TYR 892
HO
O
O
N

C

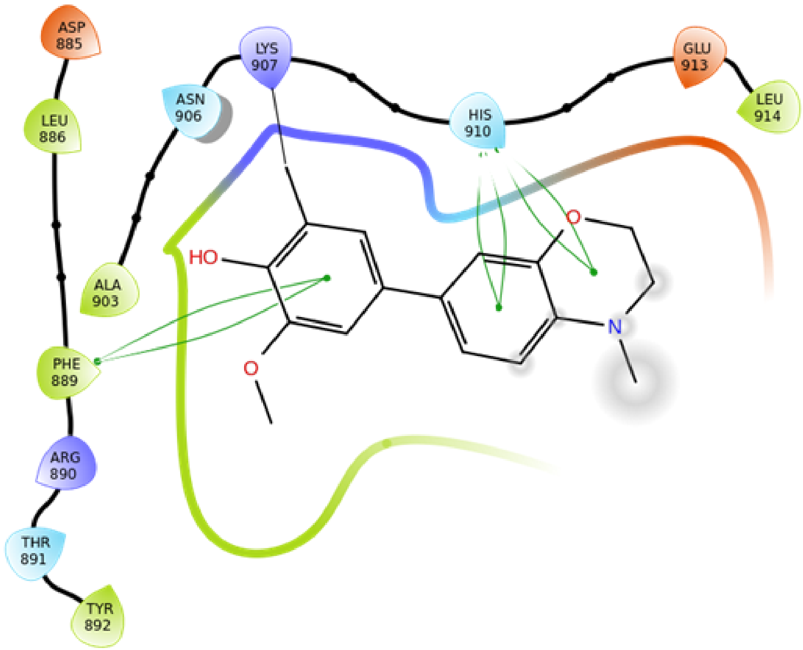

Figure S4. Schematic representation of the ligand interactions of (A) MKC9989 (PDB code: 4PL3), (B) OICR464 (PDB code: 4PL4) and (C) OICR573 (PDB code: 4PL5).

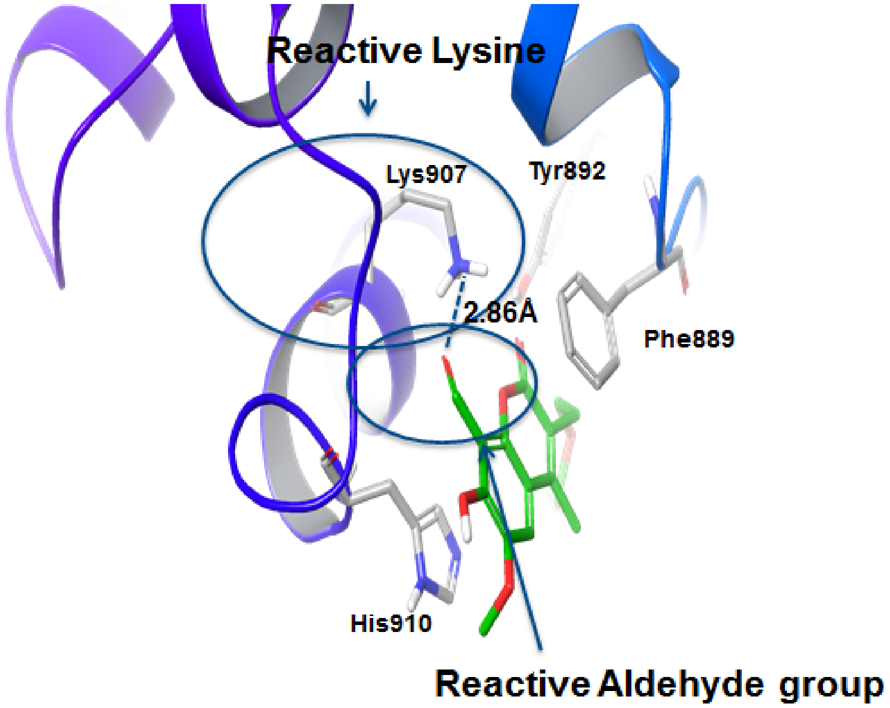

Figure S5. N-O distance between reactive moieties of the MKC9989 (aldehyde group) and reactive residue of IRE1 (Lysine 907).

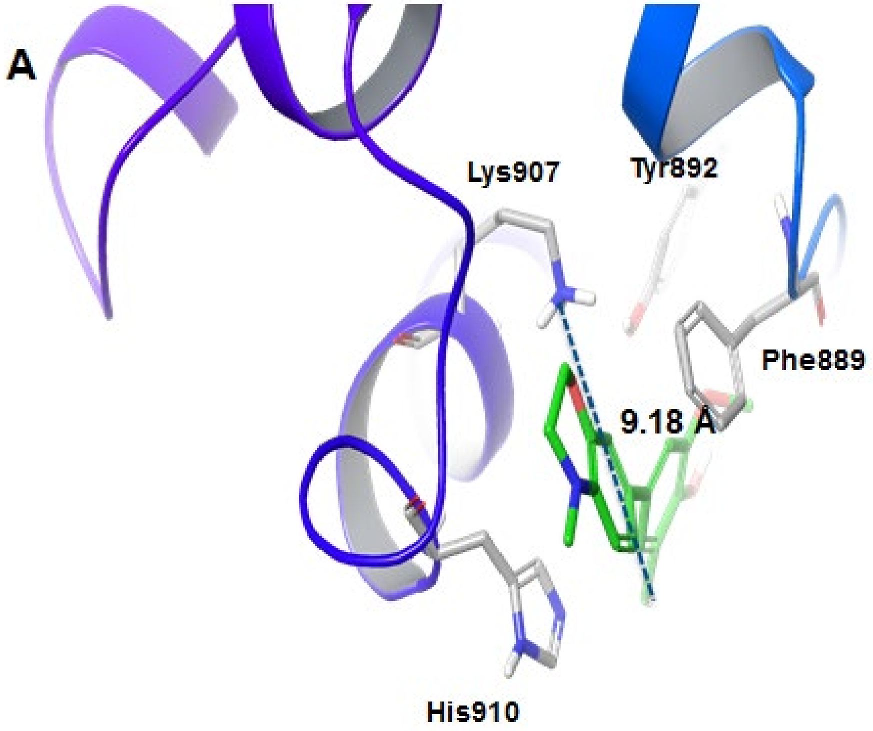
A
Lys907
Tyr892
Phe889
9.18 Å
His910

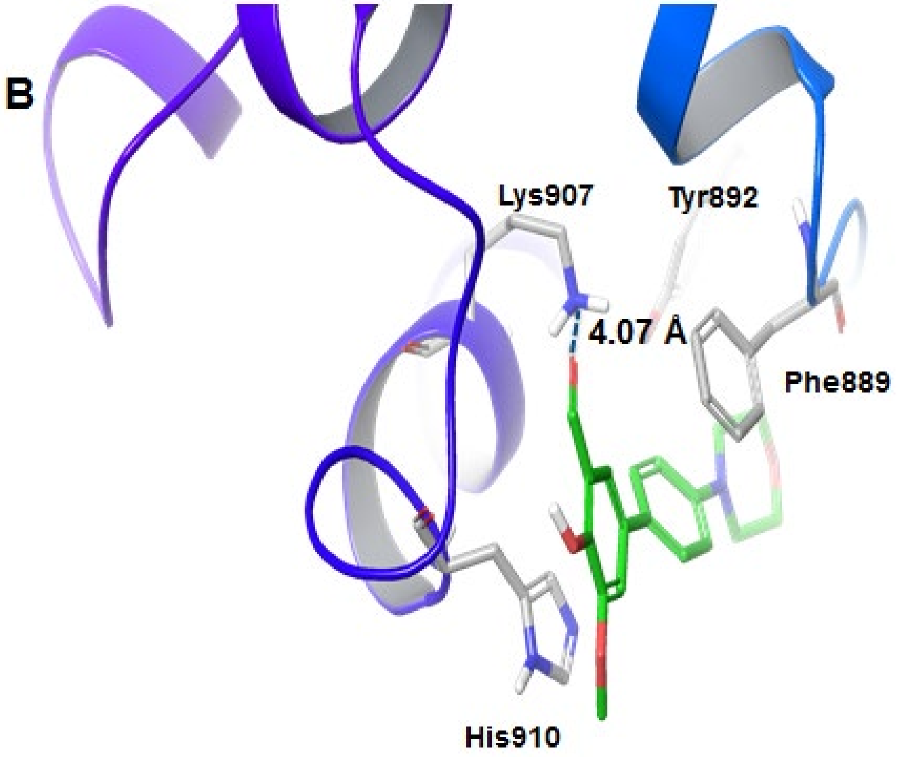
B
Lys907
Tyr892
4.07 Å
Phe889
His910

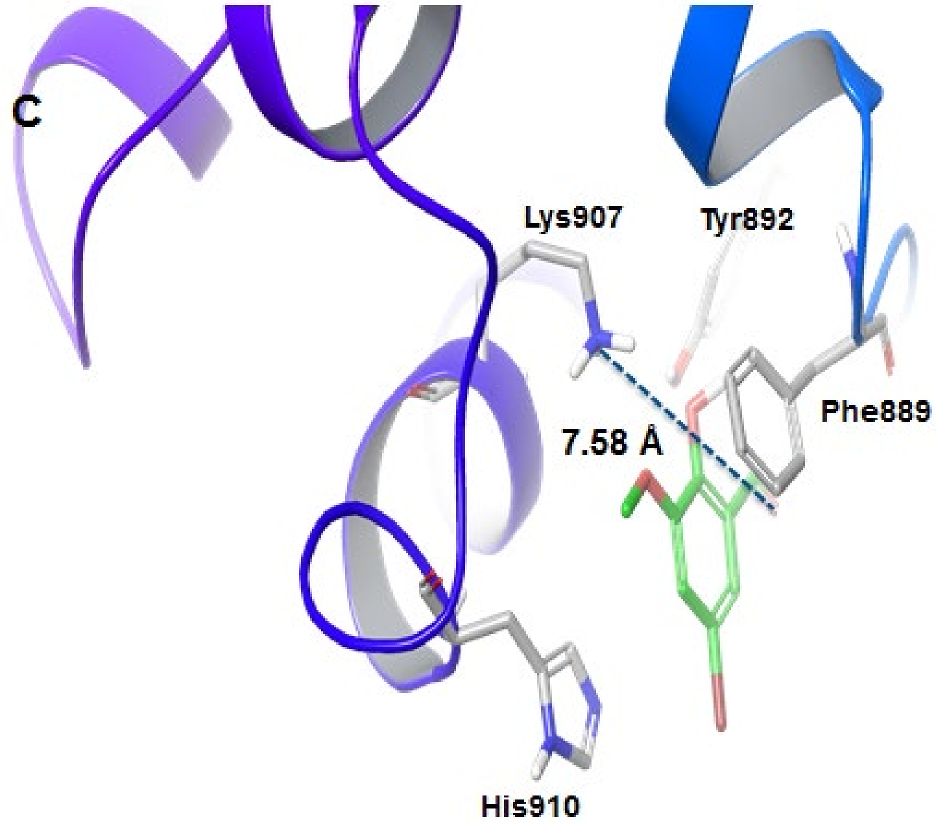

Figure S6. N-O distance between reactive moieties (Lysine 907 of IRE1 and aldehyde group of ligand) of (A) OICR464, (B) OICR573, (C) HAA3.

Table S2. N-O distance between reactive moieties of the HAA ligands (aldehyde group) and reactive residue of IRE1 (Lysine 907).

**Ligand**	**Aldehyde (O) - Lys907 (N) distance (Å)**
MKC9989	2.86
OICR464	9.18
OICR573	4.07
HAA 1	2.92
HAA 2	2.74
HAA 3	7.58
HAA 4	3.06
HAA 5	2.92
HAA 6	2.82

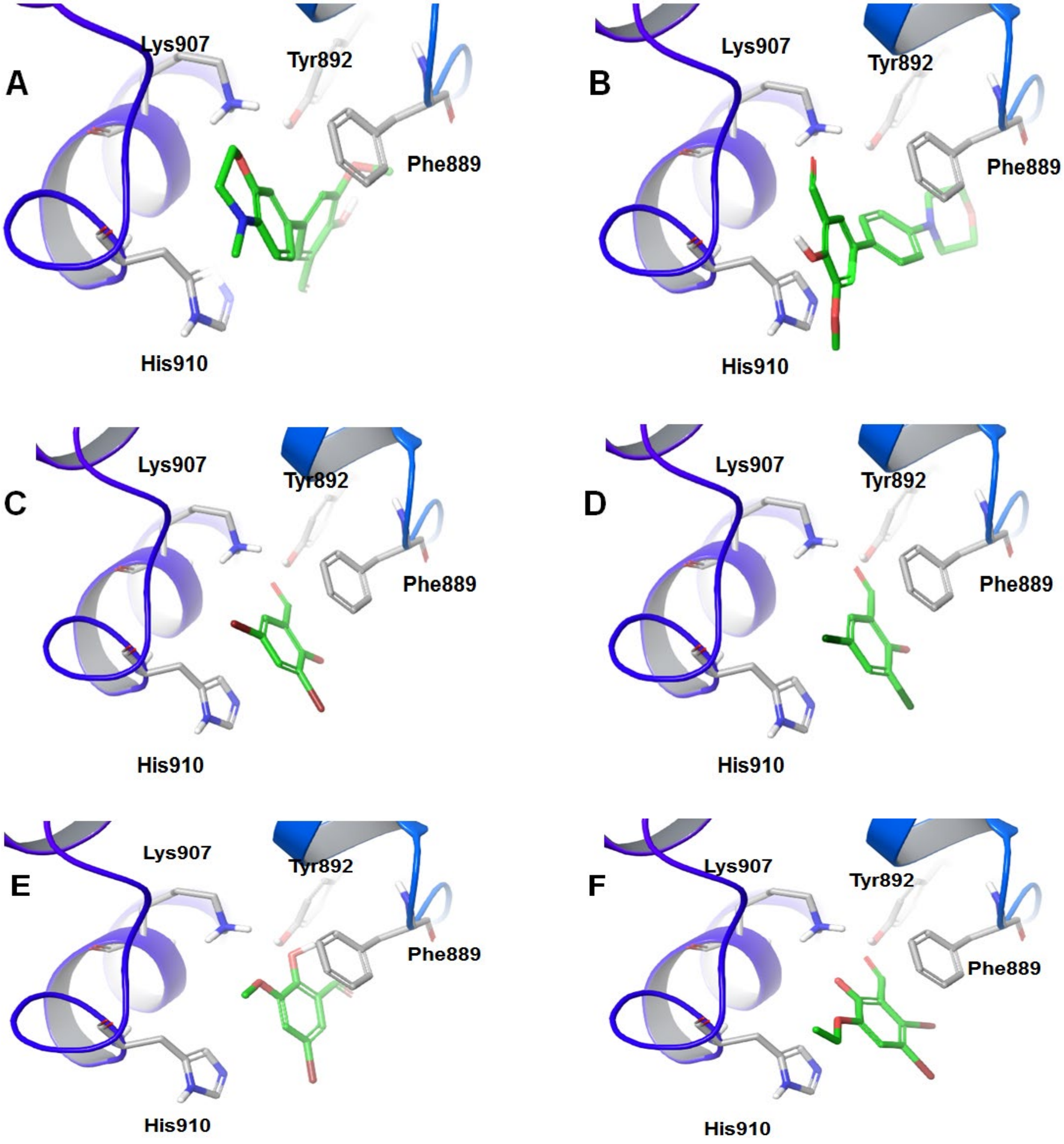
A
Lys907
Tyr892
Phe889
His910
B
Lys907
Tyr892
Phe889
His910
C
Lys907
Tyr892
Phe889
His910
D
Lys907
Tyr892
Phe889
His910
E
Lys907
Tyr892
Phe889
His910
F
Lys907
Tyr892
Phe889
His910

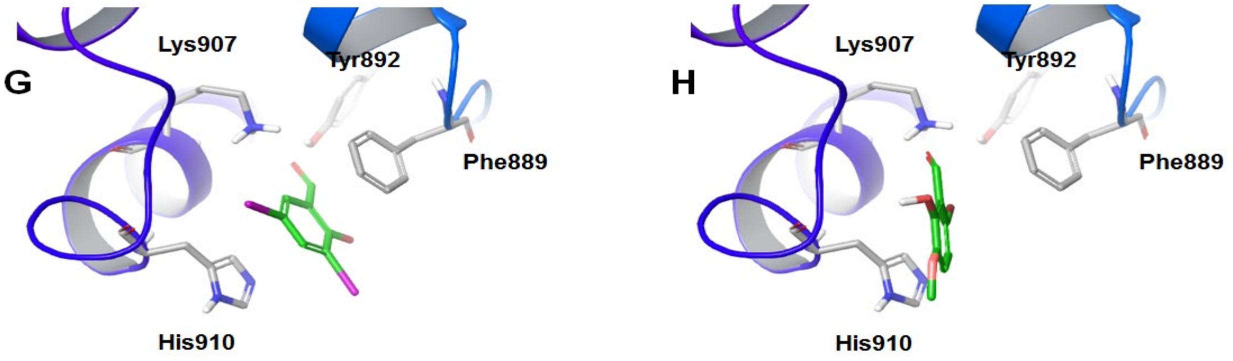

Figure S7. Predicted pose in the binding pocket of the murine RNase active site (PDB 4PL3) of (A) OICR464 ,(B) OICR573, (C) HAA1, (D) HAA2, (E) HAA3, (F) HAA4, (G) HAA5, (H) HAA6. Amino-acids involved in binding with the HAA ligands are shown in stick model.

A

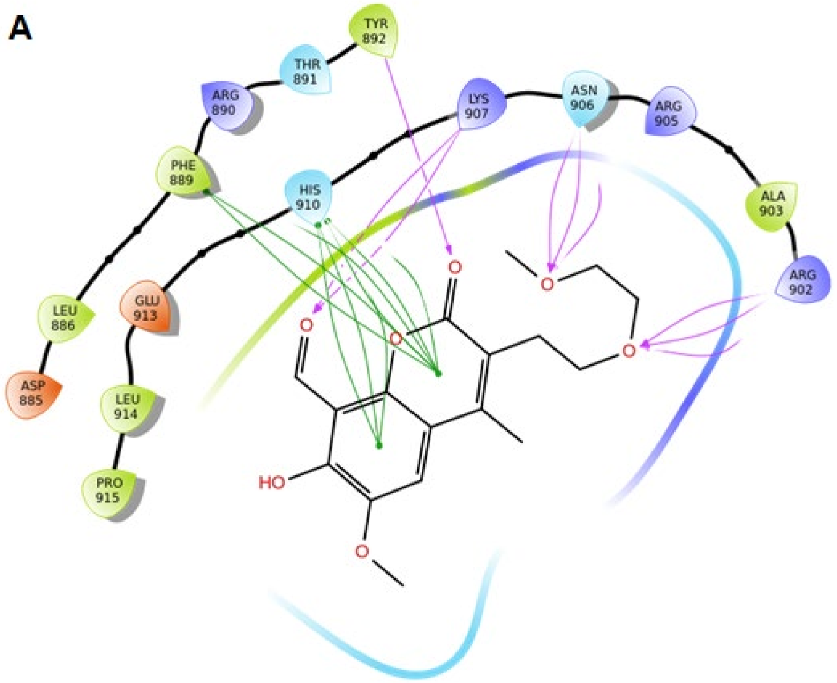
TYR 892
THR 891
ARG 890
LYS 907
ASN 906
ARG 905
PHE 889
HIS 910
ALA 903
ARG 902
LEU 886
GLU 913
ASP 885
LEU 914
PRO 915
HO

B

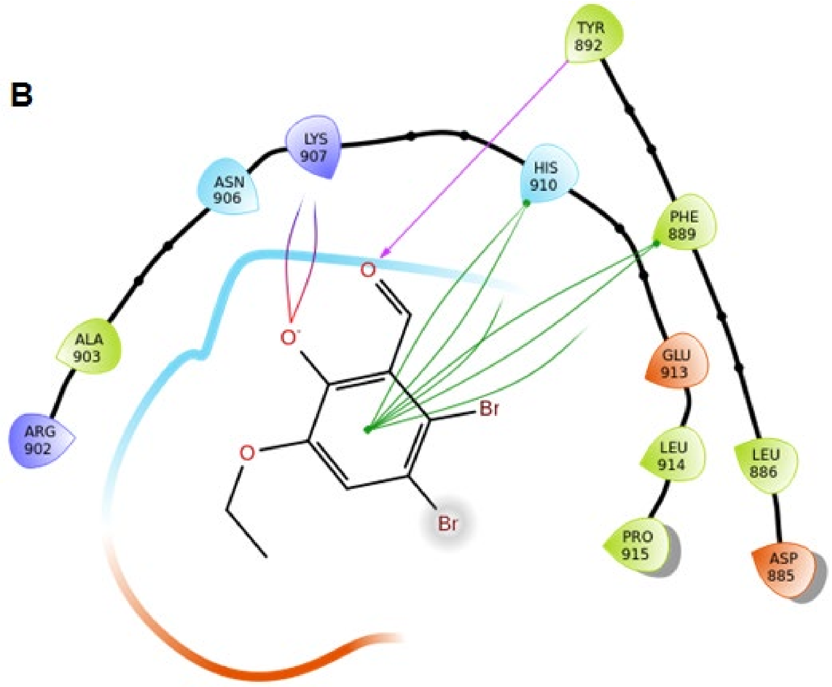
TYR 892
LYS 907
ASN 906
HIS 910
PHE 889
ALA 903
ARG 902
GLU 913
LEU 914
LEU 886
PRO 915
ASP 885
Br
Br

C

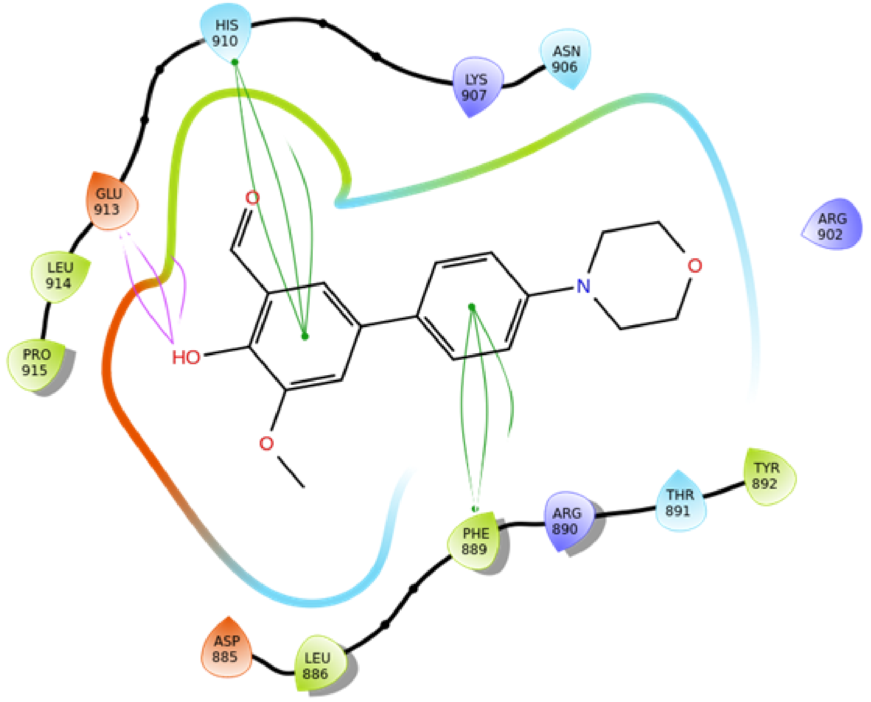

HIS 910
LYS 907
ASN 906
GLU 913
LEU 914
PRO 915
ARG 902
O
HO
N
O
O
TYR 892
THR 891
ARG 890
PHE 889
ASP 885
LEU 886

D

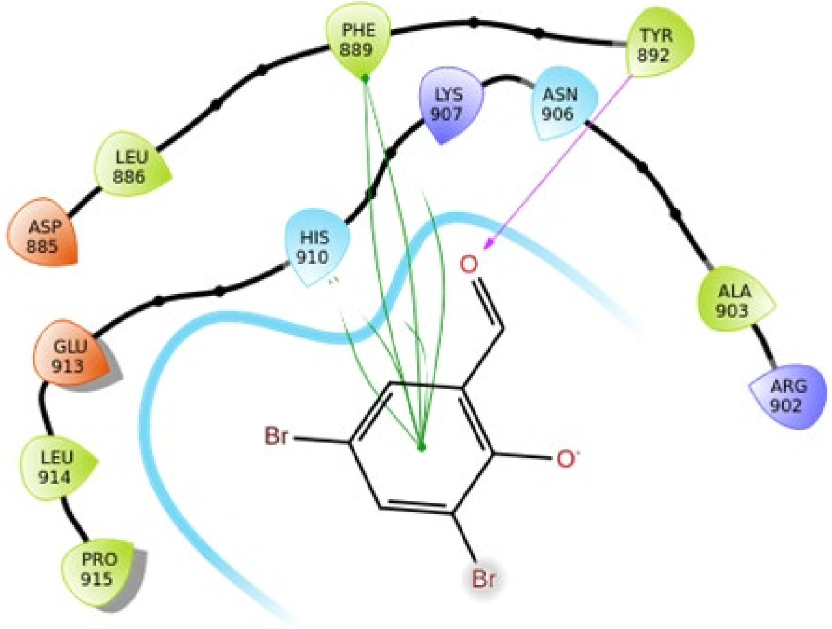

PHE 889
TYR 892
LYS 907
ASN 906
LEU 886
ASP 885
HIS 910
O
ALA 903
GLU 913
ARG 902
Br
O
LEU 914
Br
PRO 915

E

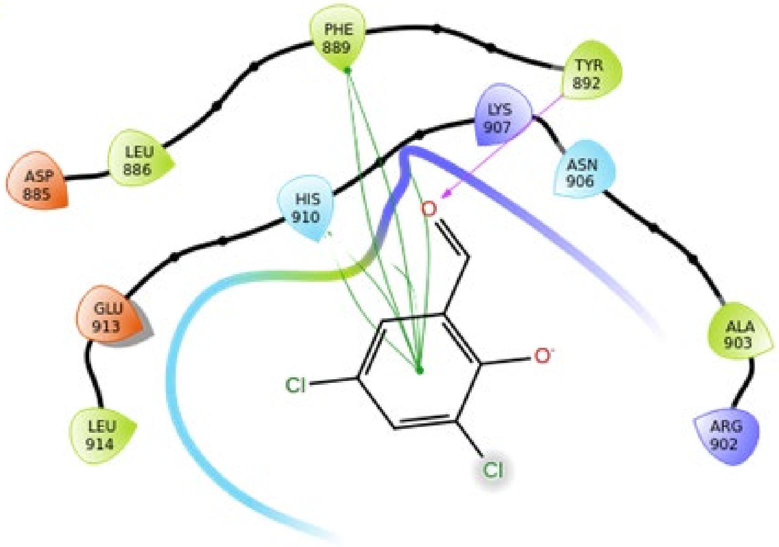

PHE 889
TYR 892
LYS 907
LEU 886
ASP 885
ASN 906
HIS 910
O
GLU 913
ALA 903
O-
Cl
LEU 914
ARG 902
Cl

F

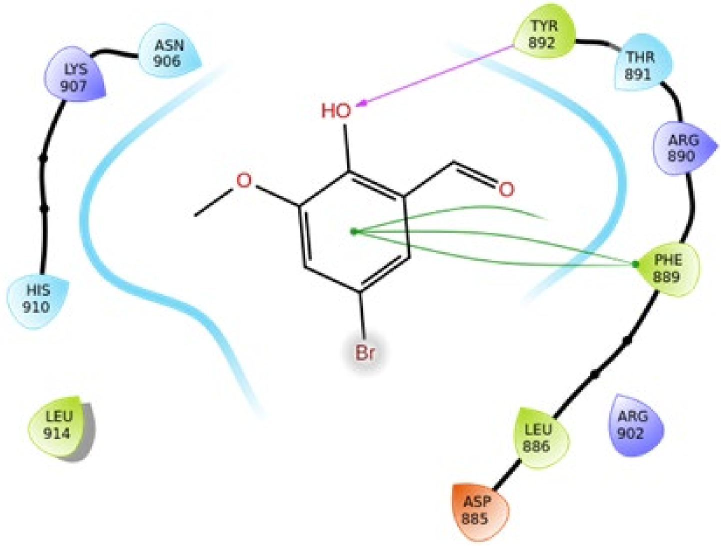

TYR 892
ASN 906
LYS 907
THR 891
HO
ARG 890
O
O
PHE 889
HIS 910
Br
LEU 914
LEU 886
ARG 902
ASP 885

G

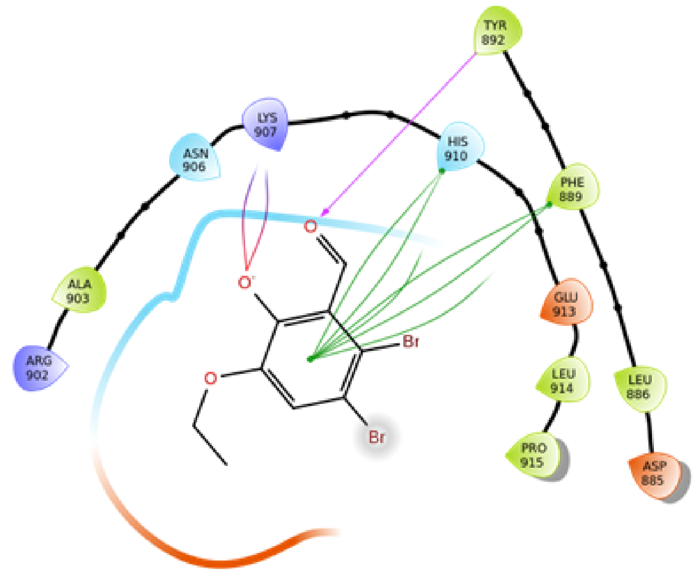
TYR
892
LYS
907
HIS
910
ASN
906
PHE
889
GLU
913
ALA
903
ARG
902
LEU
914
LEU
886
PRO
915
ASP
885
O
O⁻
O
Br
Br

H

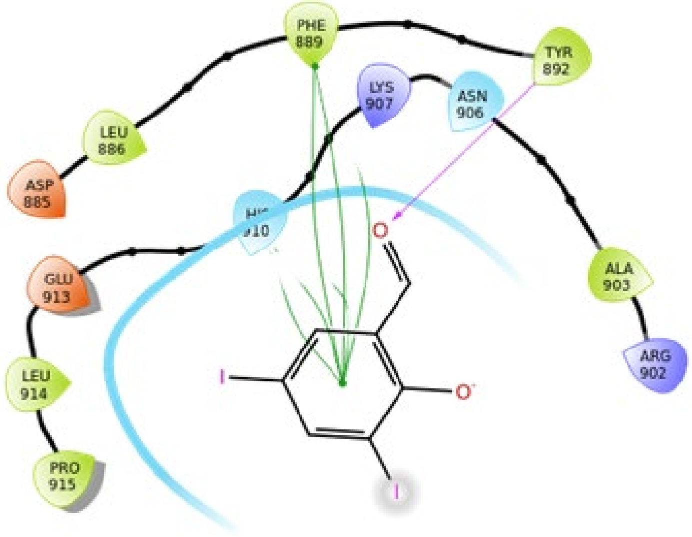
PHE
889
TYR
892
LYS
907
ASN
906
LEU
886
ASP
885
GLU
913
ALA
903
ARG
902
LEU
914
PRO
915
O
I
O⁻
I

I

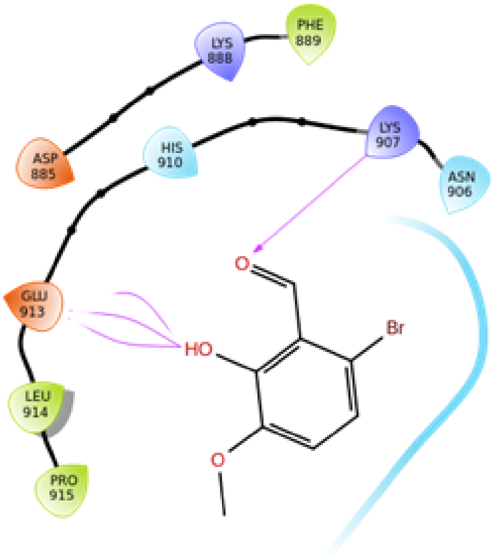

Figure S8. Schematic representations of the predicted pose in the binding pocket of the murine RNase active site (PDB 4PL3) of (A) MKC889, (B) OICR464, (C) OICR573, (D) HAA1, (E) HAA2, (F) HAA3, (G) HAA4, (H) HAA5, and (I) HAA6.

Table S3. Self-Docking Pose Prediction for available Crystal Structure Complexes

**PDB ID**	**Ligand**	**RMSD of the best predicted pose (Å) using CovDock-LO workflow**	**RMSD of the best predicted pose (Å) using CovDock-VS Workflow (default mode)**
4PL3	MKC9989	7.03	5.85
4PL4	OICR464	2.35	2.73
4PL5	OICR573	2.89	3.06

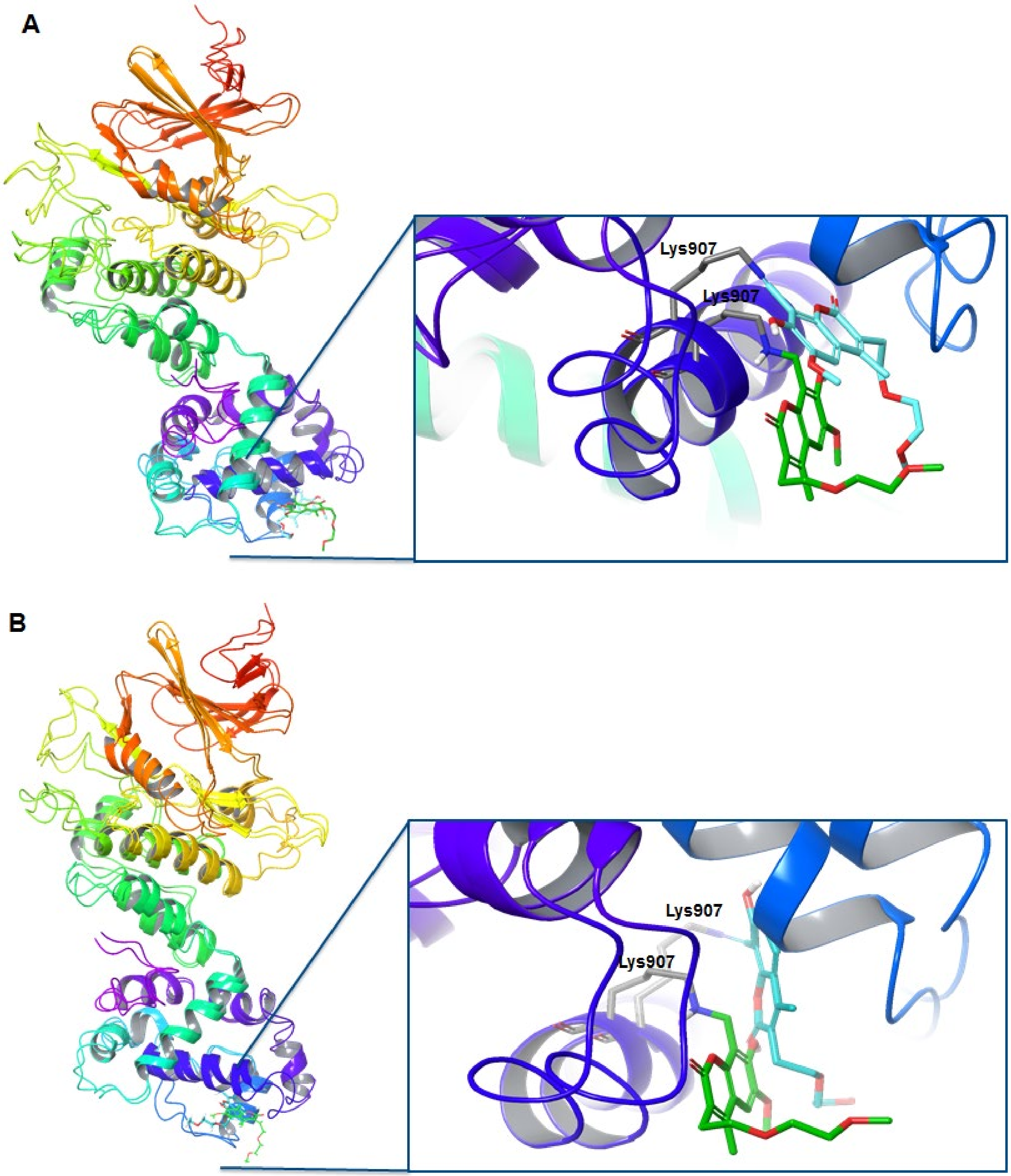
A
Lys907
Lys907
B
Lys907
Lys907

C

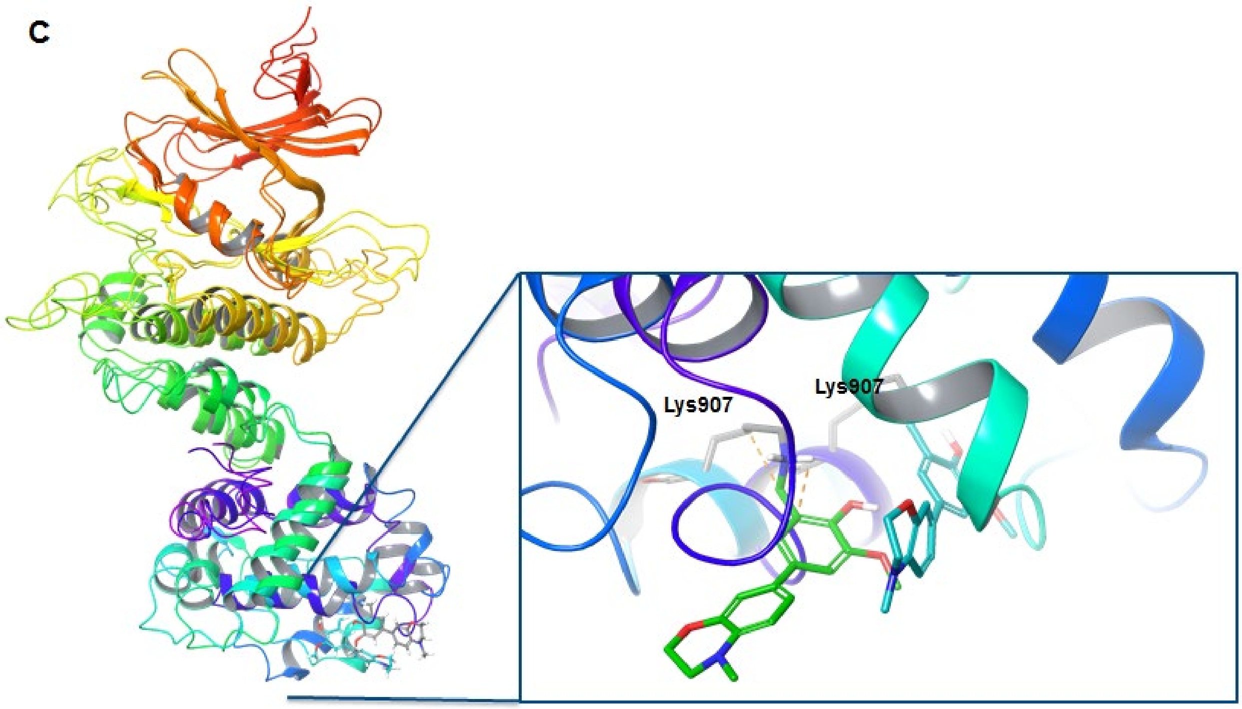

D

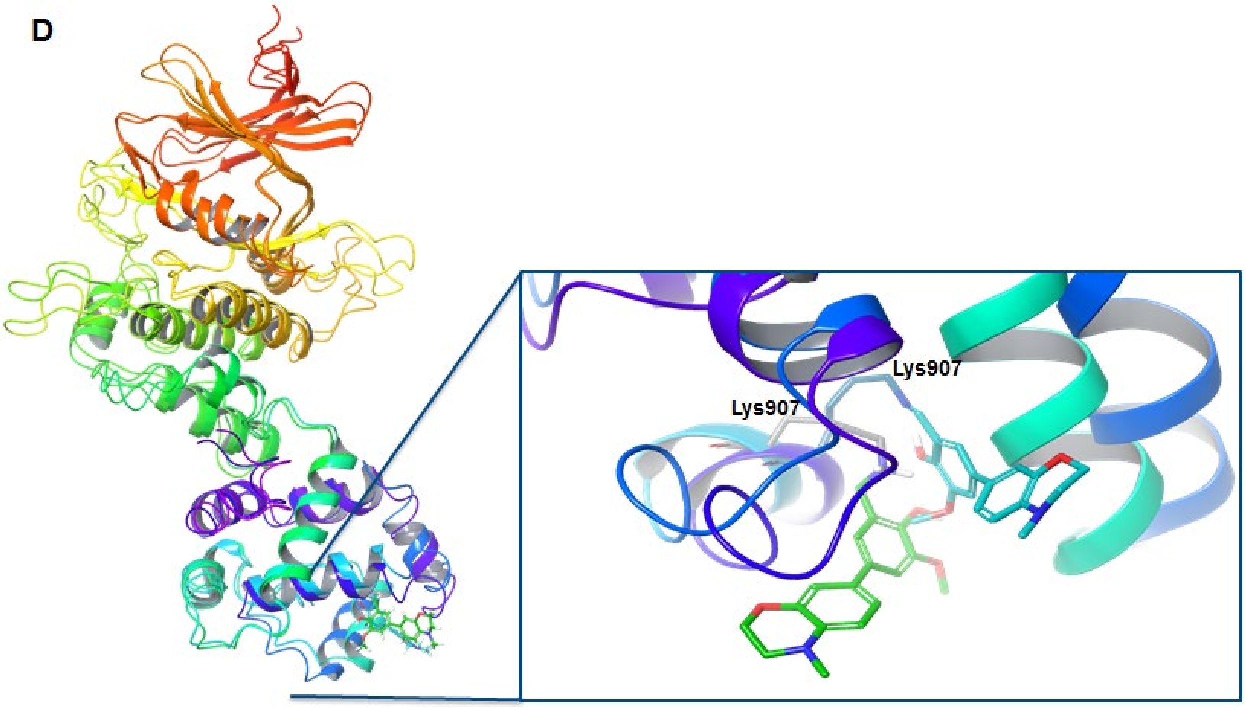

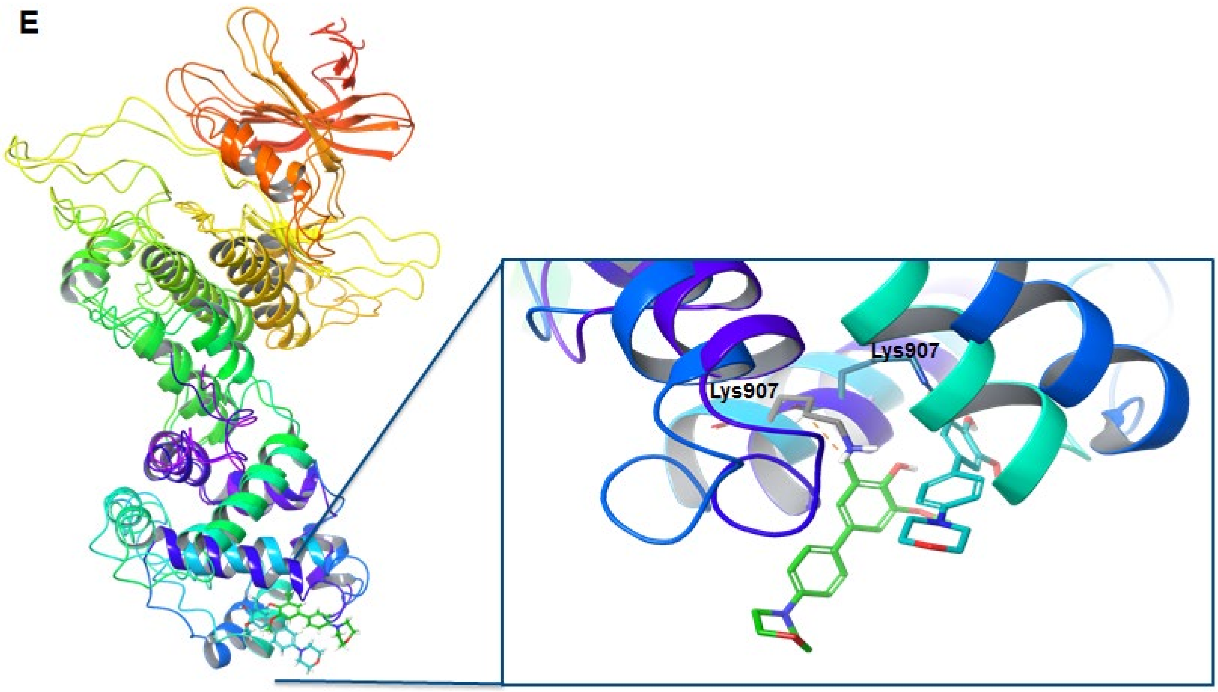

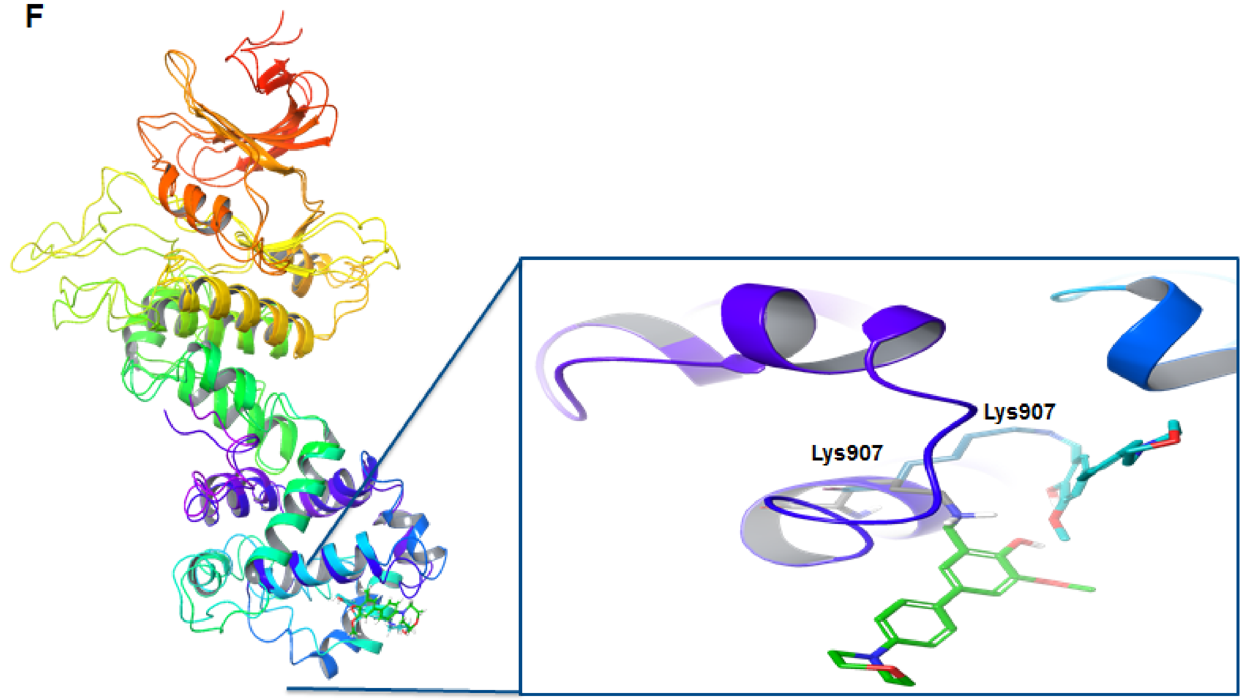

Figure S9. Superposition between the experimental crystallographic complex and the last frame of the MD simulations for docked poses generated using (A) CovDock LO and (B) CovDock VS for MKC9989 bound (PDB 4PL3); (C) CovDock LO and (D) CovDock VS for OICR464 (PDB 4PL4); (E) CovDock LO and (F) CovDock VS for OICR573 (PDB 4LP5).Lys907 is highlighted and all the other residues in the binding site are hidden for clarity.

Bibliography

1. Chenna, R. *et al.* Multiple sequence alignment with the Clustal series of programs. *Nucleic Acids Research* **31**, 3497-3500 (2003).

2. Sanches, M. *et al.* Structure and mechanism of action of the hydroxy-aryl-aldehyde class of IRE1 endoribonuclease inhibitors. *Nat Commun* **5**, 4202, doi:10.1038/ncomms5202 (2014)

3. Volkmann, K. *et al.* Potent and selective inhibitors of the inositol-requiring enzyme 1 endoribonuclease. *J Biol Chem* **286**, 12743-12755 (2011).

4. Korennykh, A. V. *et al.* Structural and functional basis for RNA cleavage by Ire1. *BMC Biol* **9**, 47 (2011).

5. Toledo Warshaviak, D., Golan, G., Borrelli, K. W., Zhu, K. & Kalid, O. Structure-based virtual screening approach for discovery of covalently bound ligands. *J Chem Inf Model* **54**, 1941-1950 (2014).

6. Zhu, K. *et al.* Docking covalent inhibitors: a parameter free approach to pose prediction and scoring. *J Chem Inf Model* **54**, 1932-1940 (2014).
